# Evolutionary plasticity in the innate immune function of Akirin

**DOI:** 10.1371/journal.pgen.1007494

**Published:** 2018-07-23

**Authors:** Jolanta Polanowska, Jia-Xuan Chen, Julien Soulé, Shizue Omi, Jerome Belougne, Clara Taffoni, Nathalie Pujol, Matthias Selbach, Olivier Zugasti, Jonathan J. Ewbank

**Affiliations:** 1 Aix Marseille Univ, CNRS, INSERM, CIML, Marseille, France; 2 Max Delbrück Center for Molecular Medicine, Berlin, Germany; 3 Charité-Universitätsmedizin Berlin, Berlin, Germany; The University of Texas Health Science Center at Houston, UNITED STATES

## Abstract

Eukaryotic gene expression requires the coordinated action of transcription factors, chromatin remodelling complexes and RNA polymerase. The conserved nuclear protein Akirin plays a central role in immune gene expression in insects and mammals, linking the SWI/SNF chromatin-remodelling complex with the transcription factor NFκB. Although nematodes lack NFκB, Akirin is also indispensable for the expression of defence genes in the epidermis of *Caenorhabditis elegans* following natural fungal infection. Through a combination of reverse genetics and biochemistry, we discovered that in *C*. *elegans* Akirin has conserved its role of bridging chromatin-remodellers and transcription factors, but that the identity of its functional partners is different since it forms a physical complex with NuRD proteins and the POU-class transcription factor CEH-18. In addition to providing a substantial step forward in our understanding of innate immune gene regulation in *C*. *elegans*, our results give insight into the molecular evolution of lineage-specific signalling pathways.

## Author summary

When animals are infected, as part of their innate immune response, they switch on defence genes that encode proteins that help fight pathogens. We use the nematode *Caenorhabditis elegans* to understand the steps in this process. When infected, *C*. *elegans* can turn on clusters of antimicrobial peptide genes. We have discovered that the coordinated expression of these genes requires a particular chromatin remodelling complex (proteins that open up compact DNA), working in conjunction with a protein called Akirin. Akirin plays a central role in immune gene expression in insects and mammals, but we found that although it has conserved its role of bridging chromatin-remodellers and transcription factors needed for gene expression, the identity of its functional partners is different. Our findings represent a major advance in our understanding of innate immune gene regulation in *C*. *elegans*, and give insight into how biological mechanisms can evolve.

## Introduction

A fundamental part of innate immune responses is the regulated expression of defence genes. In both vertebrates and many invertebrates, including *Drosophila*, two of the key regulators controlling innate immunity are the Rel-homology domain (RHD) protein NF-κB and its protein partner IκB [1]. Across many species, NF-κB functions in concert with members of the conserved Akirin family (InterPro: IPR024132) to govern the expression of defence genes [2]. More specifically, in vertebrates, Akirin2 bridges NF-κB and the SWI/SNF chromatin-remodelling complex, by interacting with IκB-ζ and the BRG1-Associated Factor 60 (BAF60) proteins, downstream of Toll-like receptor (TLR) signalling [3, 4]. In insects, an equivalent complex (including Relish and the Brahma-associated proteins BAP55 and BAP60 in *Drosophila*) governs antimicrobial peptide (AMP) gene expression upon infection by Gram-negative bacteria [4–6].

Infection of *Caenorhabditis elegans* by its natural pathogen *Drechmeria coniospora* [7] provokes an increase of AMP expression, but in the absence NF-κB and independently of the single TLR gene *tol-1* [8, 9]. It was therefore surprising that *akir-1*, the sole nematode Akirin orthologue was identified in a genome-wide RNAi screen for genes involved in the regulation of *nlp-29* [10, 11], an AMP gene that has been extensively used as a read-out of the epidermal innate immune response (e.g. [12–16]).

These previous studies have revealed surprising molecular innovation in the pathways that regulate AMP gene expression. To give one example, in other animal species, STAT-like transcription factors function in concert with Janus kinases (JAKs). But in *C*. *elegans*, although there are no JAKs [17], the 2 STAT-like proteins, STA-1 and STA-2, function in antiviral [18] and antifungal immunity [19], respectively. In the latter case, STA-2’s function appears to be modulated by a nematode-specific member of the SLC6 family, SNF-12, acting downstream of the GPCR DCAR-1 and a p38 MAPK pathway to regulate *nlp-29* expression [20]. Here, we undertook a focused study of *akir-1*, to understand how AMP gene expression is governed and also to gain insight into the evolution of lineage-specific signalling pathways. We have been able to identify Akirin’s functional partners in *C*. *elegans* and thus reveal an unexpected molecular swap at the core of innate immune gene expression.

## Results

### The *C*. *elegans* Akirin homolog is required for antifungal innate immunity

**Fig 1:**
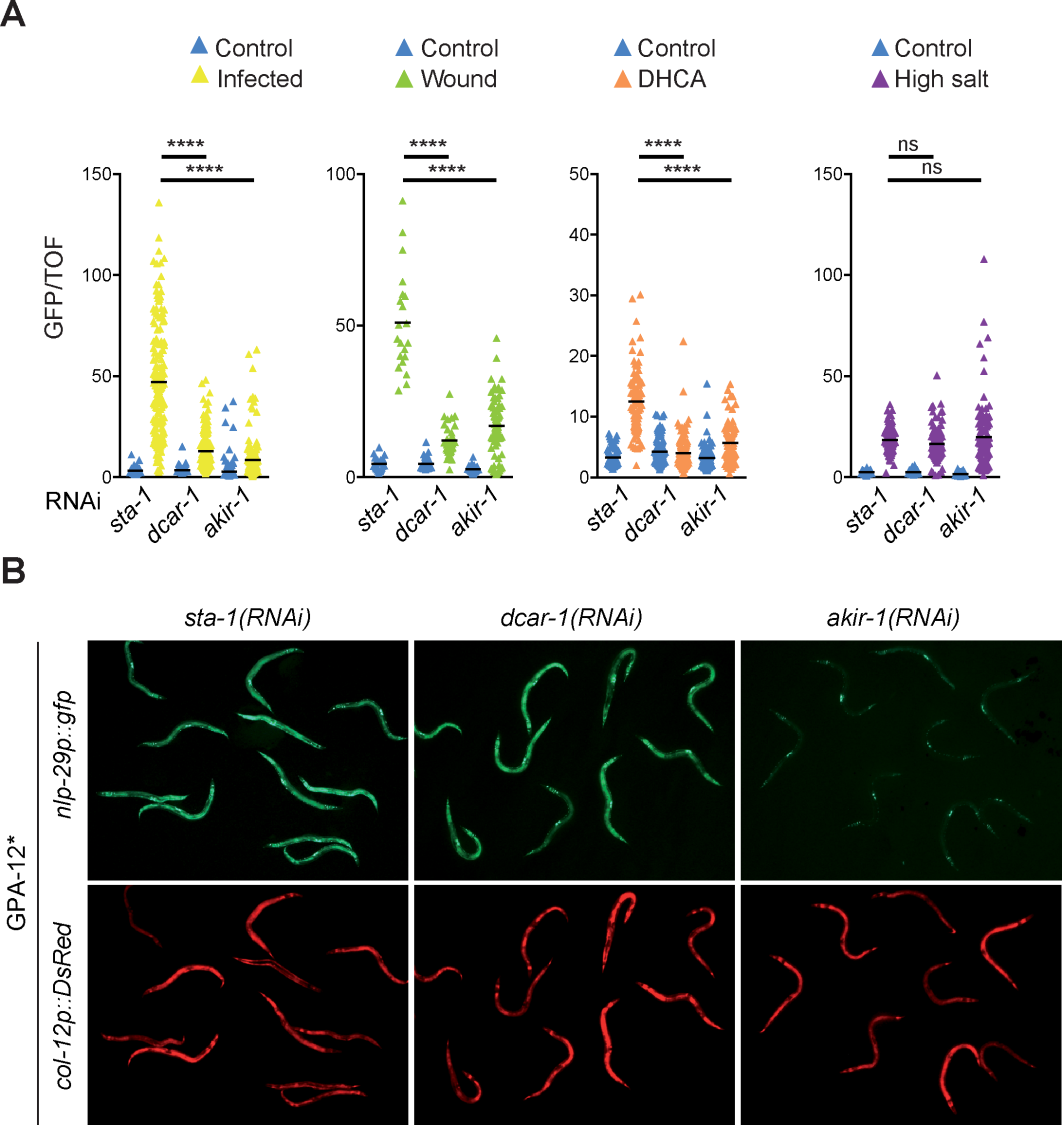
Akirin acts downstream of Gα to regulate the expression of *nlp-29*. **A.** Ratio of green fluorescence (GFP) to size (time of flight; TOF) in IG274 worms carrying the integrated array *frIs7* (containing *nlp-29p*::*gfp* and *col-12p*::*DsRed*) treated with RNAi against negative and positive controls (*sta-1*, *dcar-1*, respectively) or *akir-1* and infected by *D*. *coniospora* (Infected), wounded (Wound), treated with a 5 mM dihydrocaffeic acid solution (DHCA) or exposed to 300mM NaCl (High salt). Here and in subsequent figures representing Biosort results, unless otherwise stated, graphs are representative of at least 3 independent experiments. The black bar represents the mean value for (from left to right), n = 189, 164, 179, 184, 183, 169; 23, 21, 30, 29, 36, 68; 97, 86, 114, 111, 104, 94; 96, 85, 100, 150, 129, 113; **** p<0.0001, ns p>0.05, Dunn’s test. **B.** Fluorescent images of adult worms carrying *frIs7*, expressing a constitutively active Gα protein, GPA-12*, in the epidermis and treated with RNAi against the indicated genes. Almost all of the residual GFP expression seen upon *akir-1*(RNAi), most prominent in the vulval muscle cells, comes from *unc-53Bp*::*gfp* used as a transgenesis marker.

We previously conducted a semi-automated genome-wide RNAi screen [10] for genes that control the expression of the AMP reporter gene *nlp-29p*::*gfp*, following infection of *C*. *elegans* with *D*. *coniospora* [11]. In the screen, *sta-1* was used as a negative control since its inactivation has no observable effect on *nlp-29* reporter gene expression [19, 20]. The candidates identified as positive regulators are collectively referred to as Nipi genes, for No Induction of Peptide expression after Infection. While *akir-1*(RNAi) caused a robust reduction in the induction of *nlp-29p*::*gfp* expression after infection (Fig 1A), it did not significantly affect the size of treated worms, nor the expression of the control *col-12p*::*DsRed* reporter transgene (S1A Fig), identifying it as Nipi gene and suggesting that it could have a specific function in innate immunity. When we used an available deletion allele, *akir-1*(*gk528*), which is predicted to be a molecular null, we recapitulated the effect on *nlp-29p*::*gfp* expression (S1B Fig). This analysis was, however, hampered by the mutants’ pleiotropic phenotypes [21], including a developmental delay and very marked decrease in the expression of the control reporter transgene (S1C Fig). To avoid these confounding effects, and since RNAi of *akir-1* gave robust and reproducible results, we used *akir-1*(RNAi) for our subsequent analyses.

The induction of *nlp-29p*::*gfp* expression upon *D*. *coniospora* infection is correlated to the infectious burden, which in turn reflects the propensity of spores to bind the worm cuticle [11, 22]. There was no reduction in spore adhesion following *akir-1*(RNAi) (S1D Fig). Many genes required for the induction of *nlp-29p*::*gfp* expression after infection, including the GPCR gene *dcar-1* [20] and the STAT transcription factor-like gene *sta-2* [19], are also required for the transcriptional response of *C*. *elegans* to physical injury. We found that *akir-1*(RNAi) also abrogated reporter transgene expression upon wounding (Fig 1A). One trigger for the epidermal innate immune response is the increase in the tyrosine metabolite HPLA that accompanies infection with *D*. *coniospora*. HPLA acts via DCAR-1 to activate a p38 MAPK signalling cascade [20]. This GPCR can also be activated by the HPLA tautomer DHCA [23], a non-physiological ligand, which we use routinely as it is somewhat more potent and less toxic for worms than HPLA [20]. The induction of *nlp-29p*::*gfp* expression upon exposure to DHCA was greatly reduced upon *akir-1*(RNAi), to a degree that was comparable to *dcar-1*(RNAi) (Fig 1A). Together, these results suggest that *akir-1* is required for the activation of the epidermal innate immune response, downstream of DCAR-1.

In contrast to the induction of *nlp-29p*::*gfp* provoked by infection, wounding or DHCA, the induction of *nlp-29p*::*gfp* observed after 6 hours exposure to moderate osmotic stress is DCAR-1 and p38 MAPK PMK-1-independent [20, 24]. We found that *akir-1*(RNAi), like *dcar-1*(RNAi), did not affect the induction of reporter gene expression upon osmotic stress (Fig 1A). Unlike *dcar-1*(*RNAi*), but similar to *sta-2(RNAi)* [13, 25], *akir-1(RNAi)* abolished the strong expression of *nlp-29p*::*gfp* seen in worms expressing a constitutively active form of the Gα protein GPA-12 (GPA-12*) (Fig 1B). Together, these results support the specific role for *akir-1* in innate immune signalling, placing it downstream of, or in parallel to, *gpa-12*.

### *akir-1* likely acts in the epidermis to modulate AMP expression

**Fig 2:**
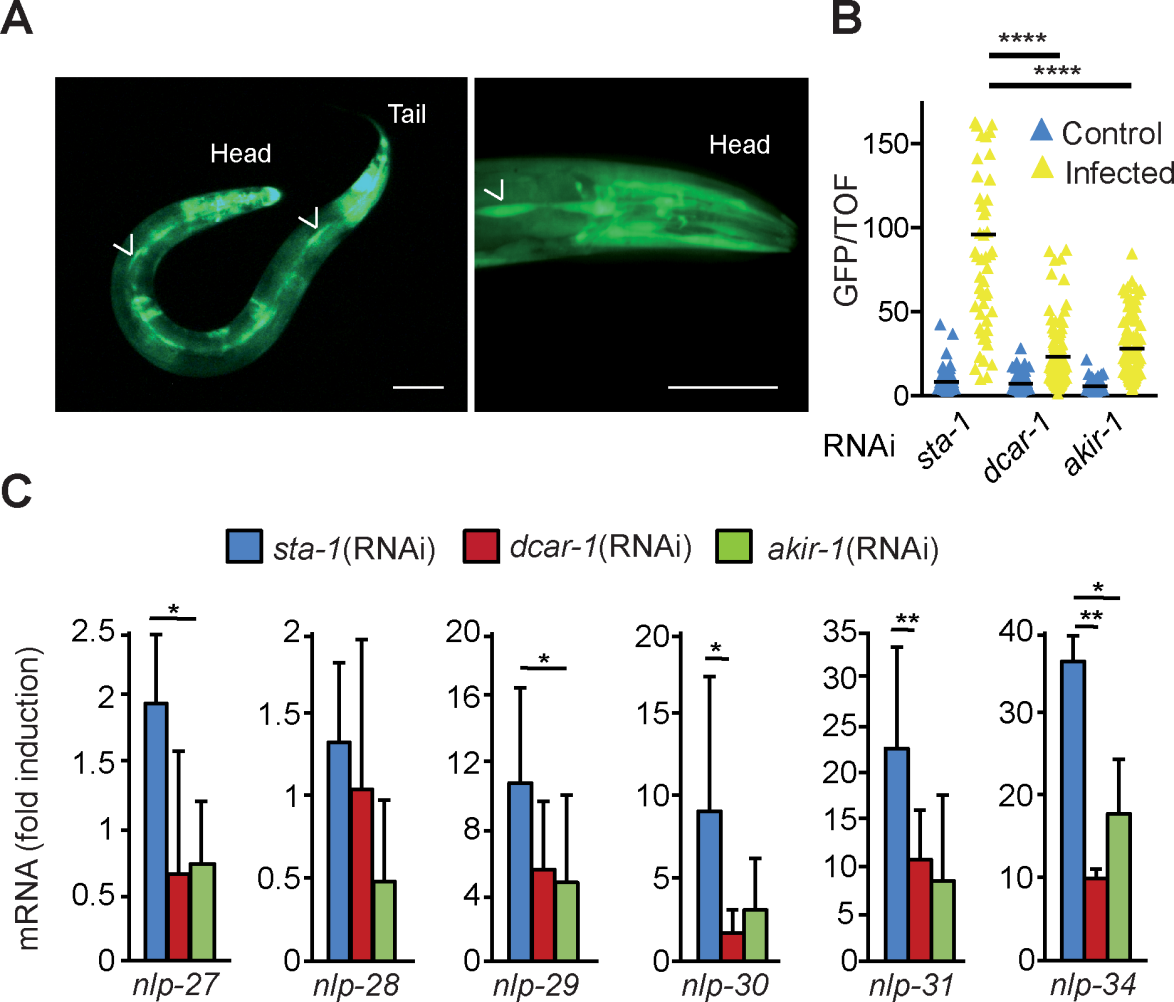
Akirin regulates multiple *nlp* genes in the epidermis. **A.** Confocal images of IG1485 transgenic worms expressing an *akir-1p*::*gfp* reporter gene showing epidermal and neuronal expression of GFP. The lateral epithelial seam cells are indicated by the arrowheads. Much of the fluorescence in the head and tail comes from neurons, seen more clearly in the right panel. Scale bar 50 μm. **B.** Ratio of green fluorescence (GFP) to size (TOF) in *rde-1*(*ne219*);*wrt-2p*::*RDE-1* worms that are largely resistant to RNAi except in the epidermis, carrying the array *frIs7*, treated with RNAi against the indicated genes and infected by *D*. *coniospora*. The black bar represents the mean value for (from left to right), n = 135, 49, 155, 102, 130, 94; **** p<0.0001, Dunn’s test. **C.** Quantitative RT-PCR analysis of the expression of genes in the *nlp-29* cluster in *rde-1*(*ne219*); *wrt-2p*::*RDE-1* worms treated with RNAi against the indicated genes and infected by *D*. *coniospora*; results are presented relative to those of uninfected worms. Data (with average and SD) are from three independent experiments (S2B Fig). **, p < 0.001; *, p < 0.01; 1-tailed ratio paired t test.

To evaluate when and where *akir-*1 was expressed, we generated strains carrying a transcriptional reporter gene (*akir-1p*::*gfp*). Consistent with previous studies [26], expression of GFP was observed from the late embryo stage onwards, peaking at the late L4 stage. Expression was most evident in the lateral epithelial seam cells, the major epidermal syncytium, hyp7, as well as in multiple head and tail neurons (Fig 2A). The different components of the p38 MAPK pathway, including *dcar-1*, *gpa-12* and *sta-2*, act in a cell autonomous fashion in the epidermis [13, 19, 20]. To determine whether this was also the case for *akir-1*, we knocked down its expression in the epidermis, using the previously characterized strain IG1502 [11, 20]. This greatly decreased *nlp-29p*::*gfp* expression upon infection, and also, as judged by qRT-PCR substantially reduced the induction of all the genes of the *nlp-29* locus, while not affecting their constitutive expression (Figs 2B, 2C & S2A and S2B). Although low levels of RNAi silencing in non-epidermal tissues have been reported for the strain JM43 [27] from which IG1502 was derived, overall our results suggest that *akir-1* acts in a cell autonomous manner in the epidermis to modulate AMP gene expression upon infection.

**Fig 3:**
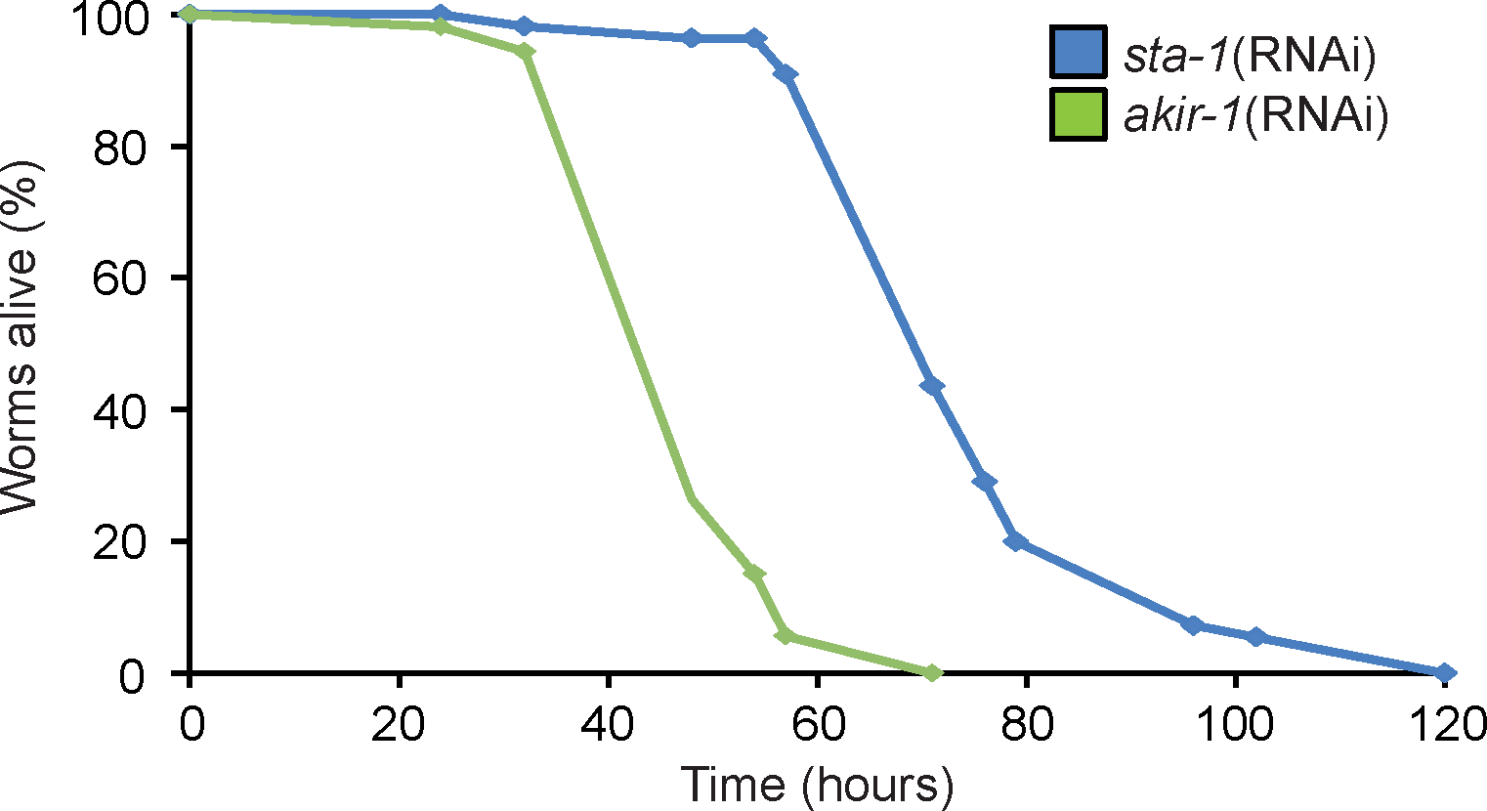
Akirin expression in the epidermis regulates resistance to fungal infection. Survival of *rde-1*(*ne219*);*wrt-2p*::*RDE-1* worms treated with RNAi against *sta-1* (negative control; n = 50) or *akir-1* (n = 50), infected with *D*. *coniospora* and cultured at 15°C. The difference between the *sta-1(RNAi)* and *akir-1(RNAi)* animals is highly significant (p<0.0001; one-sided log rank test). Data are representative of three independent experiments.

To test the functional relevance of these observations, we assayed the effect of *akir-1*(RNAi) on the resistance of *C*. *elegans* to *D*. *coniospora* infection. Compared to the negative control, *sta-1*(RNAi), knocking down *akir-1* principally in the epidermis (with strain IG1502) was associated with a significant reduction in survival (Fig 3). Interpretation of this result is complicated by the fact that the same RNAi treatment also caused a significant decrease in longevity on non-pathogenic *E*. *coli* (S2C Fig), so the reduced resistance to *D*. *coniospora* infection is not likely to result solely from the observed diminution in AMP gene expression.

### *akir-1* acts with NuRD and MEC chromatin remodelling complexes to modulate AMP expression

**Fig 4:**
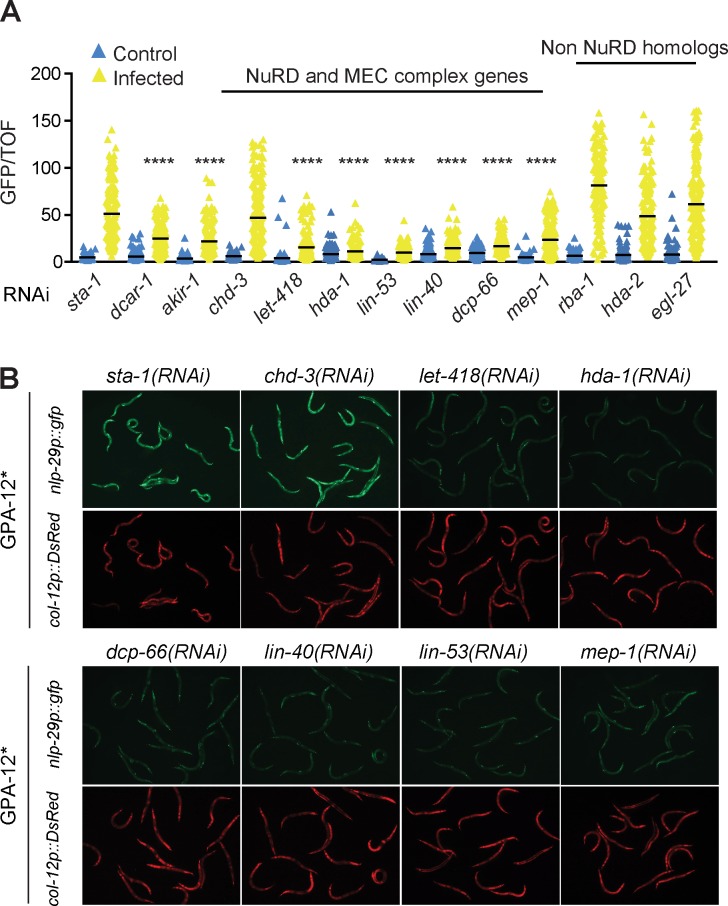
LET-418 NuRD and MEC complexes regulate *nlp-29* gene expression. **A.** Ratio of green fluorescence (GFP) to size (TOF) in worms carrying *frIs7*, treated with RNAi against control *(sta-1*, *dcar-1*, *akir-1)*, NuRD and MEC complex component, and non-NuRD chromatin remodelling component genes, and infected or not with *D*. *coniospora*. A minimum of 130 worms was used for each experiment. The black bar represents the mean value; **** p<0.0001 upon infection, relative to *sta-1*(RNAi), Dunn’s test; for the other conditions there is not a significance decrease. **B.** Fluorescent images of adult worms carrying *frIs7* and expressing GPA-12* in the epidermis and treated with RNAi against the indicated genes. See legend to Fig 2 for more details.

AKIR-1 is a member of the Akirin family. While invertebrates generally have just one Akirin protein, vertebrates can have up to 8 [28]. In mice and humans there are 2 paralogues [29]. AKIR-1 is much more similar to murine Akirin2 than Akirin1 (32.8% vs 10.9% overall sequence identity by BLASTP). While Akirin1 has been proposed to be involved in muscle regeneration and cell chemotaxis [30], as mentioned above, Akirin2 has a conserved function controlling innate immune gene expression through its interaction with BAF60/BAP60 and more generally the SWI/SNF chromatin-remodelling complex [2, 3, 5]. We therefore used RNAi to knock down the expression of components of the nematode SWI/SNF chromatin-remodelling complexes, but also of the Nucleosome Remodelling and histone Deacetylase (NuRD) and MEC complexes, as well as related genes [31]. With the exception of *swsn-1*, which caused pleiotropic development defects and affected expression of the control *col-12p*::*DsRed* reporter transgene, consistent with our previous results [11], none of the other SWI/SNF genes appeared to be required for *nlp-29p*::*gfp* expression (S3A and S3B Fig). On the other hand, knocking down 6 genes *dcp-66*, *hda-1*, *let-418*, *lin-40*, *lin-53*, and *mep-1*, largely, and specifically, blocked the expression of *nlp-29p*::*gfp* upon *D*. *coniospora* infection (Figs 4A, S3C and S3D). Of note, the 3 RNAi clones that gave the most robust Nipi phenotype, those targeting *hda-1/HDAC*, *lin-40/MTA* and *dcp-66/p66*, had been identified in the previous genome-wide screen [11]. These 3 genes encode core subunits of the two canonical chromatin-remodelling (NuRD) complexes in *C*. *elegans*. The two complexes also share LIN-53/RbAp, but differ in their Mi-2 orthologs, having either LET-418 or CHD-3. LET-418 but not CHD-3, can interact with the Krüppel-like protein MEP-1 in a distinct complex, the MEC complex [31, 32]. Our results suggest that both the LET-418-containing NuRD complex and the MEC complex are involved in defence gene expression. The 6 RNAi clones also strongly abrogated the elevated expression of *nlp-29p*::*gfp* normally seen in worms expressing GPA-12*, in clear contrast to *chd-3*(RNAi) (Fig 4B). RNAi with the same 6 clones also blocked the induction of reporter gene expression in the strain IG1502 (S4A Fig). Under these conditions, (i.e. RNAi principally in the epidermis), the induction of expression of 5 endogenous *nlp* AMP genes normally provoked by *D*. *coniospora* infection was also severely compromised (S4B Fig). In contrast, there was no evidence for a role for HDA-2, RBA-1 or EGL-27 (Fig 4A), the respective homologues of the core subunits HDA-1, LIN-53 and LIN-40, that do not form part of either of the 2 biochemically characterized NuRD complexes in *C*. *elegans* [31]. Together these results suggest that both the LET-418 containing NuRD complex and the MEC complex act cell autonomously in the epidermis, downstream of (or in parallel to) *gpa-12*, to control *nlp* AMP gene expression upon *D*. *coniospora* infection. Further, they suggest that in contrast to what has been described in flies and mammals, AMP gene expression is not dependent upon the SWI/SNF complex in *C*. *elegans* and raised the possibility that AKIR-1 might function together with the NuRD and MEC chromatin remodelling complexes.

### AKIR-1 forms a complex with components of the NuRD and MEC chromatin remodelling complexes and CEH-18

**Fig 5:**
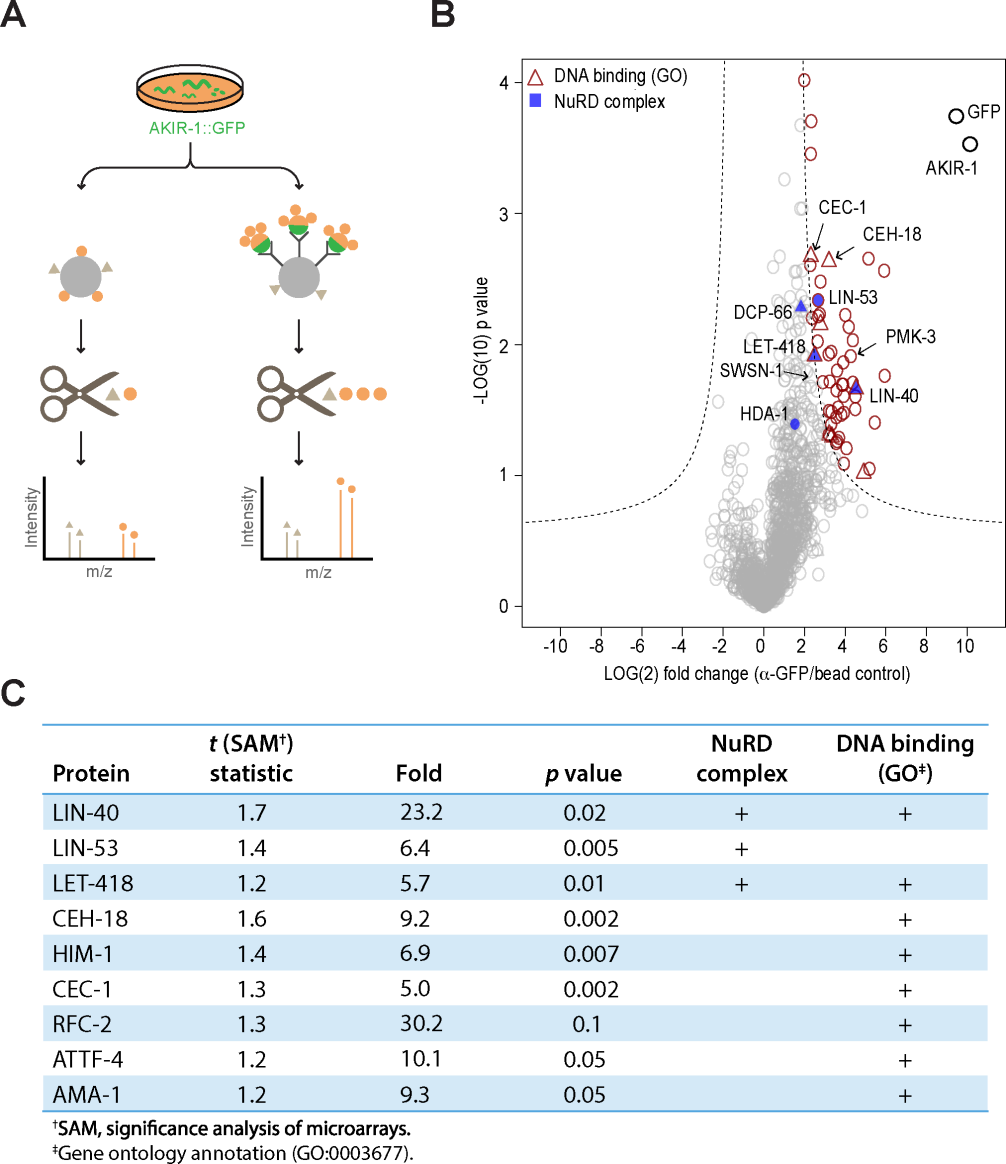
AKIR-1 interactors identified by label-free quantitative immunoprecipitation. **A.** Experimental design. Protein extracts from mixed-stage worms expressing AKIR-1::GFP were incubated with anti-GFP conjugated or control resins before proteolytic release of peptides from the immunoprecipitated proteins. The relative abundance of co-precipitated proteins was assessed by mass spectrometry. **B.** Volcano plot showing specific interaction partners (in red) of AKIR-1::GFP. The mean values for fold change from 3 independent experiments are shown. The SAM (significance analysis of microarrays) algorithm was used to evaluate the enrichment of the detected proteins. Proteins that met the combined enrichment threshold (hyperbolic curves, *t*_*0*_ = 1.2) are colored in red. Proteins with the gene ontology annotation “DNA-binding” (GO:0003677) are depicted as triangles. Known members of the NuRD complex are shown in blue. **C.** NuRD complex and/or DNA-binding proteins among the 53 high confidence AKIR-1::GFP interaction partners.

To address this possibility, we took an unbiased biochemical approach to identify the *in vivo* protein partners of AKIR-1. From a mixed-stage population of worms carrying a functional *akir-1p*::*AKIR-1*::*gfp* construct (S5 Fig), we pulled down AKIR-1::GFP by immunoprecipitation from whole worm extracts and subjected the purified proteins to mass spectrometry analysis (Fig 5A). Remarkably, all of the proteins that make up the NuRD and MEC complexes were found, i.e. LIN-40, LIN-53, LET-418, HDA-1, MEP-1 and DCP-66. The first 3, together with 6 other known or putative DNA-binding or transcription-related proteins [33], including CEH-18, were among the 53 high confidence protein partners (Fig 5B and 5C). Significantly, 9 of these 53 candidates (p = 2.7x10^-7^), again including LIN-40 and CEH-18, had been identified in our previous RNAi screen for regulators of *nlp-29p*::*gfp* [11]. In the complete list of close to 1400 protein partners, there were a further 111 hits (S1 Table), so overall, fully 35% of the known candidate regulators of AMP gene expression (Nipi genes) were recovered through this independent biochemical approach when one includes the lower confidence candidates.

When we compared the list of 53 high confidence candidate AKIR-1 binding proteins with the 190 proteins identified as potential interactors of the nematode BAP60 homologue SWSN-2.2 [34], we found only 3 common proteins, none of which have been characterized as being specific regulators of *nlp-29p*::*gfp* expression (i.e. found as Nipi genes [11]; S2 Table). Using a less stringent list of 190 potential AKIR-1 binding proteins extended the overlap to 11 common partners, with just 2 corresponding to Nipi genes (*arp-1* and *dlst-1* that encode an actin-related protein, and a predicted dihydrolipoyllysine succinyltransferase, respectively). The 11 common proteins did, however, also include SWSN-1 and SWSN-4 (S2 Table). This suggests that in some contexts, but not during its regulation of AMP gene expression, AKIR-1 might interact with the SWI/SNF complex. This functional dichotomy was further reinforced by examining the genes differentially regulated following knockdown of both *swsn-2*.*2* and its paralogue *ham-3* [34]. There were only a very small number (33/1521) of genes characterized as up-regulated by *D*. *coniospora* infection and among them, there were none encoding AMPs (S2 Table). Together these results support the idea that there is a specific AKIR-1-containing protein complex involving the NuRD and MEC chromatin remodellers, required for AMP gene regulation.

**Fig 6:**
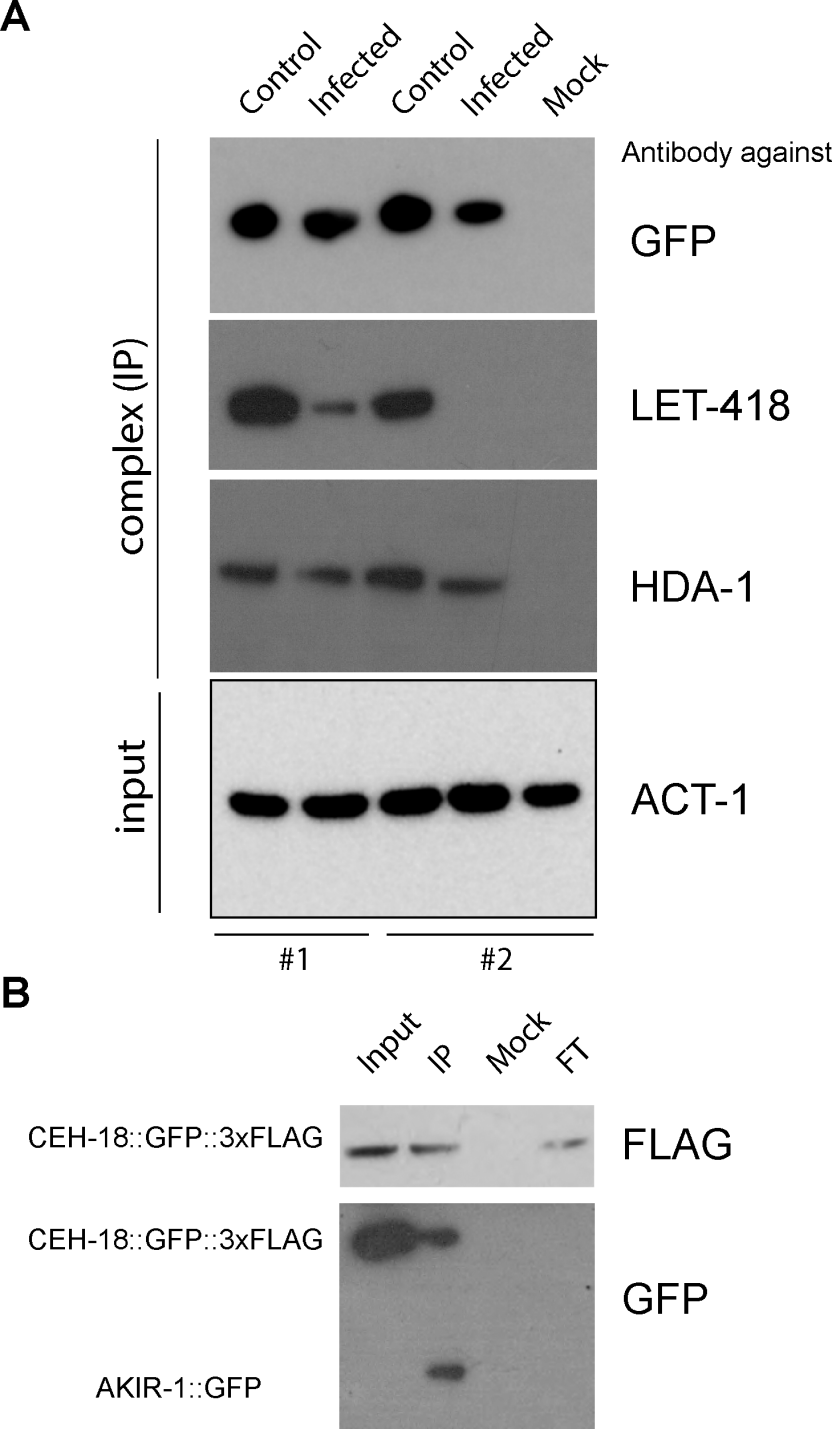
Validation of AKIR-1 interactors by Western blotting. **A.** Complexes immunopurified using an anti-GFP antibody from control or infected worms with a single copy *AKIR-1*::*GFP* insertion (*wt; frSi12[pNP157(akir-1p*::*AKIR-1*::*GFP)] II)* were probed with specific antibodies. The results for two independent pull-downs are shown. The presence of HDA-1 and LET-418 (NuRD complex components) could be confirmed. Anti-ACT-1 was used to control the total input for each immunoprecipitation. **B.** Complexes immunopurified using an anti-FLAG antibody, from a strain co-expressing AKIR-1::GFP and FLAG-tagged CEH-18 (*wt; frSi12[pNP157(akir-1p*::*AKIR-1*::*GFP)] II; wgIs533[CEH-18*::*TY1*::*GFP*::*3xFLAG + unc-119(+)]*), were probed with anti-FLAG (top panel) and anti-GFP (bottom) antibodies. In addition to the immunopurified complex (IP), the extract before immunopurification (Input), the unbound fraction (flow-through: FT) and proteins immunopurified using an unrelated antibody (Mock) were also analysed.

We therefore focused on the interaction between AKIR-1 and these chromatin-remodelling factors. We used available antibodies to validate the NuRD and MEC complex proteins LET-418 and HDA-1 as AKIR-1-interactors. Both could be detected together with AKIR-1::GFP, in samples from infected and control worms, derived from the strain used for mass spectrometric analysis, and importantly also from a strain of worms carrying a single copy *akir-1*::*gfp* insertion in the wild-type background (Fig 6A). In the latter strain, AKIR-1::GFP exhibited an predominantly nuclear localization, including in the epidermis (S6 Fig). There was a clear reduction in the quantity of LET-418 that was pulled down with AKIR-1::GFP from the samples of infected worms compared to non-infected worms. The same tendency was observed for HDA-1. These results strongly support the existence of a physical complex between AKIR-1 and the NuRD and MEC complexes in uninfected worms that changes following infection.

Seeking to confirm the potential physical interaction between AKIR-1 and CEH-18, we made use of a strain expressing both AKIR-1::GFP and a doubly-tagged version of CEH-18 (CEH-18::GFP::3xFLAG; [35]). As the AKIR-1::GFP construct is a single-copy insert its expression is expected to be close to that of the endogenous protein; it was not detectable in the total protein extract. When we analysed the complex that was pulled-down together with CEH-18, however, we were readily able to detect AKIR-1::GFP (Fig 6B), lending further support to the proposed AKIR-1/NuRD/CEH-18 complex.

### CEH-18 and AKIR-1 have overlapping non-redundant functions

**Fig 7:**
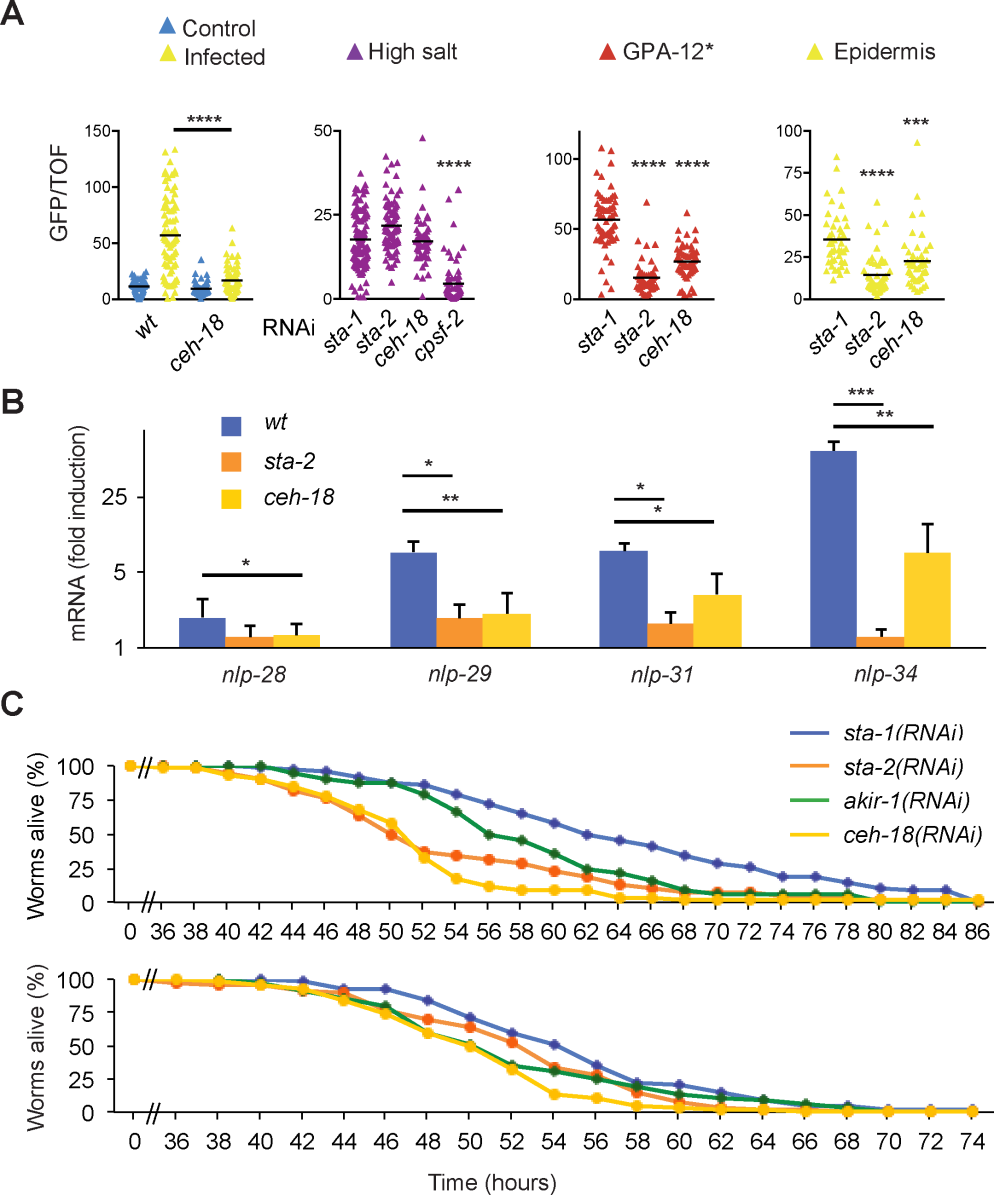
CEH-18 plays a role in host defence. **A.** Ratio of green fluorescence (GFP) to size (TOF) in wild-type (IG274) or *ceh-18*(*mg57*) mutant (IG1714) worms carrying *frIs7*, infected or not with *D*. *coniospora* for 16 h (yellow and blue, respectively; data for IG274 is as Fig 3B in [53]), and IG274 worms treated with RNAi against *sta-1* (control) or *ceh-18* and, from left to right, exposed to high salt (purple; *cpsf-2(RNAi)* is a positive control, *sta-2(RNAi)* a negative control [11]), in worms also expressing GPA-12* in the epidermis, and in the *rde-1*(*ne219*);*wrt-2p*::*RDE-1* background and infected by *D*. *coniospora*. For the latter 2 panels, *sta-2(RNAi)* is a positive control. A minimum of 45 worms was used for each condition. The black bar represents the mean value; *** p<0.001, **** p<0.0001, relative to *sta-1*(RNAi), Dunn’s test; for the other conditions there is not a significance decrease. The results of the 3 right panels are unpublished results from [11], representative of at least 4 independent experiments. **B.** Quantitative RT-PCR analysis of the expression of several genes in the *nlp-29* cluster in wild-type worms, *sta-2* and *ceh-18* mutants infected by *D*. *coniospora*; results are presented relative to those of uninfected worms. Data (with average and SD) are from three independent experiments. **, p < 0.001; *, p < 0.01; 1-tailed ratio paired t test. **C.** Results of 2 independent tests of survival of *rde-1*(*ne219*);*wrt-2p*::*RDE-1* worms treated with RNAi against *sta-1*, *sta-2*, *akir-1* or *ceh-18*, infected with *D*. *coniospora* and cultured at 25°C (n>65 for all tests). The difference between the *sta-1(RNAi)* and *ceh-18(RNAi)* animals is highly significant in both trials (p<0.0001; one-sided log rank test).

As mentioned above, we previously reported a role for *ceh*-*18* in the regulation of *nlp-29p*::*gfp* [11]; the results for 6 independent experiments assaying the effect of *ceh*-*18*(*RNAi*) on reporter gene expression following *D*. *coniospora* infection are available at http://bioinformatics.lif.univ-mrs.fr/RNAiScreen (clone sjj_ZC64.3). We were able to confirm this effect using a *ceh*-*18* mutant strain (IG1714) carrying the *frIs7* reporter gene: expression was abrogated upon infection compared to the wild-type (Fig 7A). We also demonstrated by qRT-PCR that *ceh*-*18* was required for the increased expression of several *nlp* genes after infection (Fig 7B). In common with *sta-2(RNAi)*, knocking down *ceh*-*18* by RNAi did not reduce the induction of *nlp-29p*::*gfp* provoked by osmotic stress, but did strongly abrogate the elevated reporter gene expression normally seen in worms expressing GPA-12*. RNAi against *ceh*-*18* also significantly reduced the induction of reporter gene expression in the IG1502 strain (Fig 7A). This is the same pattern of phenotypes as seen with *akir*-*1(RNAi)* (Figs 1A, 1B and 2B). The non-redundant function of CEH-18 resembles that of its binding partner, AKIR-1, supporting the hypothesis that they act together in a common complex.

When we assayed the effect of *ceh-18*(RNAi) on the resistance of *C*. *elegans* to *D*. *coniospora* infection, we observed a significant reduction in survival of IG1502 that was more pronounced than that seen upon *akir-1*(RNAi) or *sta-2*(RNAi) (Fig 7C). Unlike *akir*-*1(RNAi)* that reduces worm longevity (S2C Fig), *ceh*-*18(RNAi)* extends lifespan [36]. These results therefore support a specific role for *ceh*-*18* in innate defence against *D*. *coniospora* infection, potentially via a regulation of immune gene expression.

### AKIR-1 binds to AMP gene promoters

**Fig 8:**
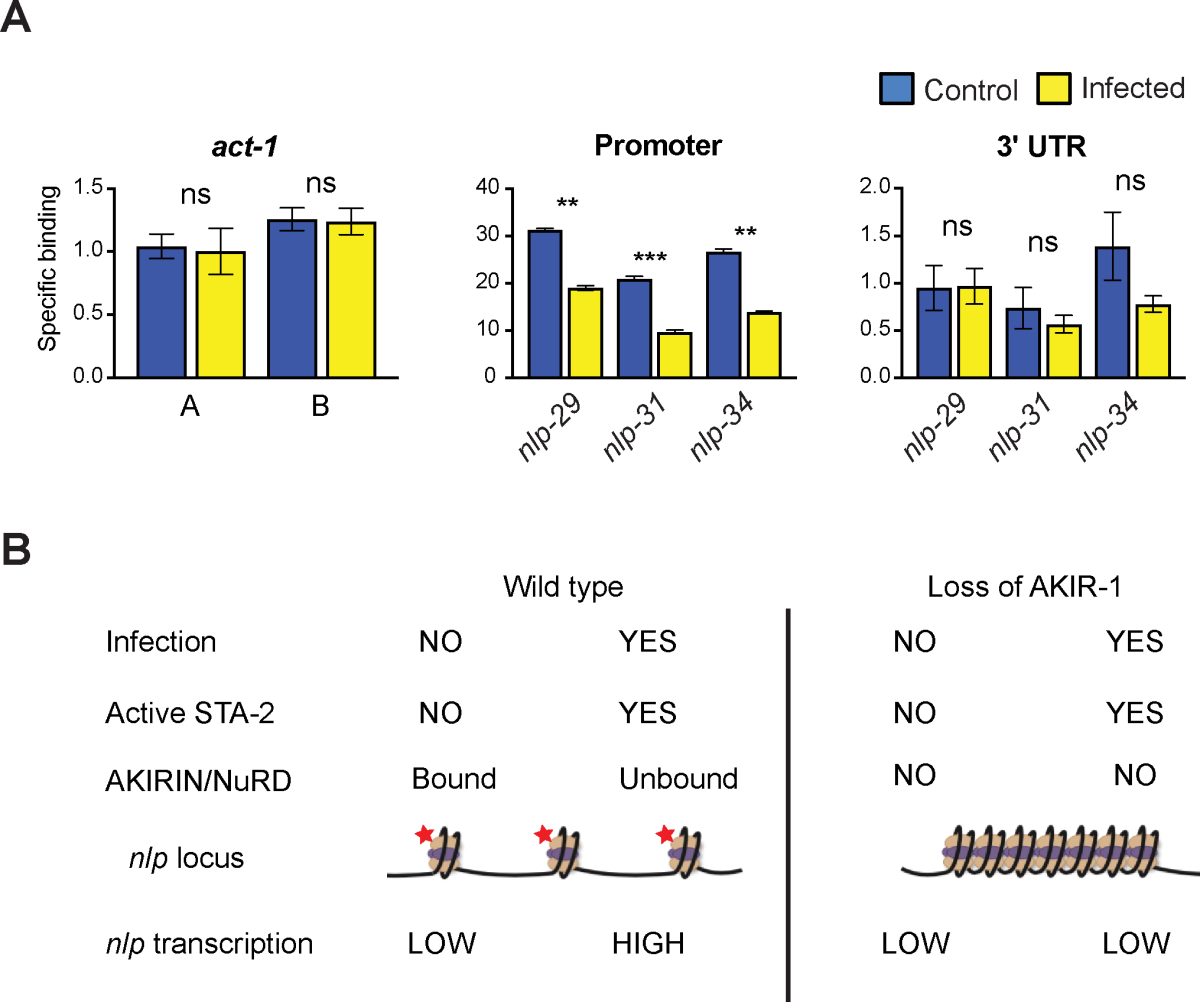
AKIR-1 binds preferentially to *nlp* gene promoters in the absence of infection. **A.** Specific binding of AKIR-1::GFP onto promoters (left panels) or 3’ UTR (right panel) of *act-1* (left panel; 2 different PCR amplicons, A and B), and *nlp-29*, *nlp-31* and *nlp-34*, represented as the fold enrichment of the specific ChIP signal obtained using an anti-GFP antibody for immunoprecipitation relative to that when blocked beads were used, measured by quantitative PCR. Data is normalised to input; the average (and standard error) from three independent experiments is shown. ***, p < 0.0001; **, p < 0.001; ns, p > 0.1; paired 2-tail Student’s t test. **B.** Model for the role of AKIR-1 in the regulation of *nlp* AMP gene expression upon infection. Under normal conditions (left), the AKIR-1/NuRD complex is recruited to the *nlp-29* locus, leading to modification (red stars) of histones (ovoids), and formation of an open chromatin structure. Upon infection, STA-2 is activated and, following removal of the AKIR-1/NuRD complex, is responsible for expression of the *nlp* genes. Infection could impact chromatin structure, but here we assume that it does not. When AKIR-1 is absent (right), an open chromatin structure cannot be formed, precluding STA-2-dependent expression of the *nlp* genes following infection, but not affecting the low basal STA-2-independent gene expression. The images are adapted, with permission, from https://www.activemotif.com.

We then addressed the question of whether AKIR-1 (and by extension CEH-18) has the potential to interact with DNA, by chromatin immunoprecipitation (ChIP), using the strain of worms carrying a single copy *akir-1*::*gfp* insertion. We first tested the specificity of the ChIP by assaying the occupancy of AKIR-1::GFP on the promoter of *act-1*, an actin-encoding gene that is used as a control for qRT-PCR since its expression is unaffected by *D*. *coniospora* infection [37]. We detected a low and constant occupancy of the *act-1* promoter using samples from uninfected or infected populations of worms (Fig 8A). We take this to reflect non-specific binding. We then assayed the capacity of AKIR-1::GFP to associate with DNA fragments corresponding to the promoters of 3 AMP genes, or to their 3’ UTRs. For all 3 AMP genes assayed, binding to the 3’ UTRs appeared to be non-specific. On the other hand, we observed markedly higher binding to the promoter regions relative to the 3’ UTRs. The 3 genes, *nlp-29*, *nlp-31* and *nlp-34* are strongly induced by *D*. *coniospora* infection [24]. There was a >10-fold higher occupancy of AKIR-1::GFP on DNA in the samples from non-infected worms relative to the infected ones (Fig 8A). Taken together, our results support a model, discussed further below, in which AKIR-1 plays 2 indissociable roles. First, in association with the NuRD and MEC complexes, it binds to the promoters of defence genes and potentially recruits transcription factors including CEH-18. Our results suggest that this does not influence the STA-2-independent basal expression of *nlp* genes. Secondly, AKIR-1 and its protein partners negatively regulate the STA-2-dependent transcription of defence genes, with this repression being relieved upon their removal from their binding sites following infection (Fig 8B). This could explain why loss of AKIR-1 (or CEH-18) function is associated with an incapacity to express AMP genes upon infection.

## Discussion

We are interested in the mechanisms involved in the regulated expression of *nlp-29*, a representative of one class of AMP genes in *C*. *elegans* [24, 38, 39]. In common with many other AMP genes, the level of *nlp-29* mRNA rapidly increases following either physical injury or infection with the nematophagous fungus *D*. *coniospora*. In both cases, the integrity of the cuticle and underlying epidermis is compromised. Although we have advanced in our understanding of how this triggers the innate immune response, and how the associated signal transduction pathway is organized, the details of the transcriptional regulation remain to be fully elucidated. We previously identified ELT-3, an epidermis specific GATA factor as being partially required, in a generic fashion, for *nlp-29* expression [24]. The STAT-like transcription factor STA-2 plays a more specific role. It is largely dispensable for the constitutive expression of *nlp-29*, but is required for its induction upon wounding and infection [13, 19]. In this work, we have made a considerable step forward by characterizing the key role of AKIR-1 and identifying its protein partners, including the NuRD and MEC complex chromatin remodelling proteins and the transcription factor CEH-18. All these factors are required for AMP gene expression after fungal infection of the nematode epidermis.

CEH-18 is a member of the POU subgroup of the Hox class of homeodomain transcription factors. These are regulators of cellular proliferation, differentiation and migration across species. In *C*. *elegans*, *ceh-18* has primarily been characterized for its negative regulatory role in a somatic gonadal sheath cell-dependent pathway that governs oocyte meiotic arrest [40]. It has not been implicated in innate immunity previously. Among POU transcription factor genes in *Drosophila*, *Dfr/Vvl*, *Pdm1/nub* and *Pdm2/miti* were identified in a screen for transcriptional regulators that bind the NF-κB-family transcription factor Dif; they are important for the control of AMP gene expression [41–43]. The corresponding proteins were not, however, identified as physical interactors of Akirin in *Drosophila* [5]. Thus if POU transcription factors do have a conserved role in regulating AMP gene expression, their precise function must have evolved, especially as nematodes lack Rel-family transcription factors [9].

Another transcription factor, LIN-40, a GATA protein, NuRD complex component and one of two *C*. *elegans* homologs of human metastasis-associated protein MTA1, was the top hit among AKIR-1’s binding partners. Recent genome-wide ChIP-seq data from the “model organism encyclopedia of regulatory networks” project (via www.encodeproject.org), revealed the presence of LIN-40 at the *nlp-29* promoter (binding site peak, V: 3984375) in DNA from uninfected young adult worms. This independent line of evidence supports the presence of a NuRD/AKIR-1 complex within this AMP gene cluster in the absence of infection. Consistent with our current understanding of its mechanism of action, we did not find STA-2 among the AKIR-1-interacting proteins. In the simplest model, a complex of AKIR-1, CEH-18 and the NuRD/MEC chromatin remodelling proteins is recruited to the *nlp* locus and opens it, but represses gene expression. Upon infection, the chromatin structure allows activated STA-2 access to the AMP gene promoters, and removal of the repressive NuRD/AKIR-1/CEH-18 complex permits gene expression. It is noteworthy that 3 of the 53 high-confidence AKIR-1 interactors are implicated in ubiquitin-mediated protein turn-over, and that in preliminary tests, *in vitro* ubiqutination activity could be detected within the purified AKIR-1 protein complex specifically after infection, not before.

Chromatin remodelling at the promoters of immune genes can prime them for enhanced activation [44]. Many AMP genes in *C*. *elegans*, as in other species, are arranged in genomic clusters [24]. AKIR-1-dependent modification of chromatin structure offers the possibility of coordinating a rapid increase in the expression of neighbouring AMP genes, potentially important when faced with a fast-growing pathogen like *D*. *coniospora*.

Akirin functions together with the SWI/SNF complex in other species. Although we excluded a role for the *C*. *elegans* SWI/SNF complex in *nlp-29* expression, we did identify some SWI/SNF complex proteins, including SWSN-1, -3, -4 and -6, among the potential AKIR-1 binding partners. These were found through an unbiased whole-organism approach; it is likely that we sampled separate complexes from different tissues. Indeed AKIR-1 is known to be expressed widely and also to have essential functions in development; it is necessary for synaptonemal complex (SC) disassembly during meiosis [21]. These different candidates therefore merit investigation in the context of AKIR-1’s other functions. It would also clearly be of interest to attempt to recover AKIR-1 interactors specifically from the epidermis, but this is a technical feat beyond the current study.

SC disassembly involves a conserved RAS/ERK (Extracellular signal-regulated kinase) MAPK cascade. Interestingly, the same pathway is required for the response of *C*. *elegans* to infection by the Gram-positive bacterium *Microbacterium nematophilum* [45]. Within the rectal epithelium, it cooperates with a Gαq signalling pathway to trigger changes in cell morphology. At the same time, in motor neurons, Gαq functions independently of RAS signalling to influence nematode behaviour in the presence of *M*. *nematophilum* [46]. Following infection, it also acts in the pharynx to regulate, non-cell autonomously, defence gene expression in the intestine [47]. These instances illustrate how the physiological response to infection is a mélange of interconnected signal transduction cascades. Further studies will be required to establish whether *akir-1* is required for any or all of these processes.

Across species, MAPKs act as regulators of chromatin structure. In yeast, the p38-related MAPK Hog1 physically interacts with the RSC chromatin-remodelling complex. This association is increased upon osmotic stress and is thought to direct the complex to bind osmo-responsive genes, changing nucleosome structure, increasing RNA polymerase II binding and causing a burst of transcription [48]. In vertebrates, the SWI/SNF subunit BAF60 can be phosphorylated by p38 MAPK, also targeting it to specific loci [49]. It is not yet clear whether PMK-1 directly phosphorylates AKIR-1 or NuRD/MEC complex proteins; it was not found as a physical interactor of AKIR-1. The p38 MAPK PMK-3 on the other hand was. RNAi of *pmk-3* does not inhibit *nlp-29* expression [11]. Notably, *pmk-3* does participate in adult axon regeneration, in a p38 pathway that while sharing some elements with the epidermal innate immune pathway [50] is clearly distinct. Our results therefore raise the possibility that AKIR-1 plays a role in axon regeneration, in association with PMK-3.

We established that the SWI/SNF complex does not play a major part in modulating AMP gene expression in the epidermis. Rather the NuRD and MEC complexes, in a physical complex with AKIR-1 and CEH-18 play an essential role. One possible cause of this evolutionary re-wiring of a regulatory circuit could be the loss of NF-κB from nematodes, which has also led to a restructuring of the TLR pathway [51]. The precise evolutionary trajectories that led to these changes can only be the subject of speculation, but these lineage-specific adaptations likely reflect the extreme selective pressure that is exerted by pathogens. This plasticity is even more remarkable when one considers the essential developmental processes that many of these factors are involved in, limiting the degree of change that can be tolerated. In conclusion, as well as substantially advancing our understanding of immune defences in *C*. *elegans*, our results illustrate how an organism can evolve novel molecular mechanisms to fight infection while conserving an overall regulatory logic.

## Materials and Methods

### Nematode strains

All strains were maintained on nematode growth media (NGM) and fed with *E*. *coli* strain OP50 [52]. The wild-type reference strain is N2 Bristol. Strains carrying *akir-1(gk528)*, *ceh-18*(*mg57*), *rde-1(ne300)* and the transgene [*ceh-18*::*TY1*::*GFP*::*3xFLAG*] (OP533) were obtained from the *Caenorhabditis* Genetics Center (CGC). Double mutants and strains containing multiple independent transgenes were generated by conventional crossing. The strains IG274 (containing *frIs*7[*nlp-29p*::*gfp*, *col-12p*::*DsRed*] *IV*) and IG1389 (containing *frIs7* and *frIs30*[*col-19p*::GPA-12*,*unc-53pB*::*gfp*] *I*) have been described elsewhere [13, 38]. We recently validated the use of strains carrying *col-19p*::GPA-12* as a model for the inductive part of the epidermal innate immune response [53].

### Constructs and transgenic lines

Full genotypes of the transgenic strains are given below. The *akir-1p*::*AKIR-1*::*gfp* construct contains 1.6 kb of genomic sequence upstream of the start codon of E01A2.6 and was obtained by PCR fusion [54] using primers JEP2091, JEP2092; JEP2108, JEP568, JEP569 and JEP570 and using genomic DNA and the vector pPD95.75 as templates. Microinjections were first performed using 20 ng/μl of the construct and the coinjection marker *myo-2p*::*mCherry* at a concentration of 80 ng/μl into N2 animals. Although transgenic strains were readily obtained, the observed fluorescence declined rapidly across successive generations (OZ unpublished observations). Since mutants in the Argonaute gene *rde-1* do not exhibit transcriptional silencing of transgenes in the soma [55], we then performed the same microinjection but used *rde-1(ne300)* animals. From three independent transgenic lines generated, one was subsequently integrated using X rays and outcrossed three times with *rde-1(ne300)* generating *IG1550 rde-1(ne300) V; frIs32[akir-1p*::*AKIR-1*::*gfp; myo-2p*::*mCherry]*. This strain maintained transgene expression constantly across multiple generations. All additional strains carrying the *frIs32[akir-1p*::*AKIR-1*::*gfp; myo-2p*::*mCherry]* transgene were obtained by conventional crosses. The *akir-1p*::*gfp* construct was generated by PCR fusions using primers: JEP2091, JEP2092, JEP2095, JEP2096, JEP569 and JEP570 using genomic DNA, and the vector pPD95.75 as templates. Microinjections were performed using 20 ng/μl of the construct of interest and the co-injection marker *pNP135* (*unc-53pB1*::*DsRed*) at a concentration of 80 ng/μl in WT animals. Three independent lines were obtained and IG1485 was retained for further study. The single copy strain IG1654 carrying *AKIR-1*::*GFP* (*wt; frSi12[pNP157(akir-1p*::*AKIR-1*::*GFP)] II)* was obtained by CRISPR in N2 worms at the location of the *ttTi5605* Mos1 insertion [56] and subsequent excision of the self-excising cassette (SEC) [57]. pNP157 was made by Gibson cloning from a vector containing the SEC and recombination arms for *ttTi5605* (pAP087, kindly provided by Ari Pani) flanking *akir-1p*::*AKIR-1*::*GFP*, amplified from the strain IG1550, and the 3’UTR of *akir-1*, amplified from the wild type strain. The full locus *akir-1p*::*AKIR-1*::*GFP*::*3’UTR_akir-1* was confirmed by sequencing (primers available upon request). Microinjections were performed using pNP157 (*akir-1p*::*AKIR-1*::*GFP*) at 10ng/μl, pDD122 (sgRNA *ttTi5605*) at 40 ng/μl (kindly provided by Ari Pani), pCFJ90 *(myo-2p*::*mCherry)* at 2.5ng/μl, pCFJ104 *myo-3p*::*mCherry* at 5ng/μl and #46168 (*eft-3p*::*CAS9-SV40_NLS*::*tbb-2* 3'UTR; Addgene) at 30 ng/μl. Roller worms that did not display red fluorescence were selected then heat shocked to remove the SEC by FloxP as described [57].

### Full genotypes of transgenic strains

IG274 *wt; frIs7[nlp-29p*::*gfp*, *col-12p*::*DsRed] IV* [24]

IG1389 *wt*; *frIs7 IV; frIs30[col-19p*::GPA-12*,*pNP21(unc-53pB*::*gfp)] I* [13]

IG1485 *wt*; *frEx547[akir-1p*::*gfp; unc-53p*::*DsRed]*

IG1502 *rde-1(ne219) V; Is[wrt-2p*::*RDE-1; myo-2p*::*mCherry]; frIs7 IV* [20]

IG1550 *rde-1(ne300) V; frIs32[akir-1p*::*AKIR-1*::*gfp; myo-2p*::*mCherry]*

IG1555 *wt; frIs32[akir-1p*::*AKIR-1*::*gfp; myo-2p*::*mCherry]*

IG1575 *akir-1(gk528) I; rde-1(ne300) V; frIs32[akir-1p*::*AKIR-1*::*gfp; myo-2p*::*mCherry]*

IG1577 *akir-1(gk528) I; frIs32[akir-1p*::*AKIR-1*::*gfp; myo-2p*::*mCherry]*

IG1654 *wt; frSi12[pNP157(akir-1p*::*AKIR-1*::*GFP)] II*

IG1665 *wt; frSi12[pNP157(akir-1p*::*AKIR-1*::*GFP)] II; wgIs533[CEH-18*::*TY1*::*GFP*::*3xFLAG + unc-119(+)]*

IG1714 *ceh-18(mg57) X*; *frIs7[nlp-29p*::*gfp*, *col-12p*::*DsRed] IV*

### PCR fusion primers

The sequences of the primers used are:

JEP568: agcttgcatgcctgcaggtcgact,

JEP569: aagggcccgtacggccgactagtagg,

JEP570: ggaaacagttatgtttggtatattggg,

JEP2091: gatgaacaccgatagagagcaactg

JEP2092: gctctcgcggaaatgacgaat

JEP2095: agtgaaaagttcttctcctttactcattttacttctgaaagaaataatttgtggtta

JEP2096: atgagtaaaggagaagaacttttcact

JEP2108: agtcgacctgcaggcatgcaagctggagaggtacgaataggaatagtcat

### RNA interference

RNAi clones were from the Ahringer [58] and the Vidal [59] RNAi libraries. Insert sequences were verified and target genes confirmed using Clone Mapper [60] before use. To limit RNAi principally to the epidermis, we used the strain IG1502 *rde-1(ne219);Is[wrt-2p*::*RDE-1; myo-2p*::*mCherry];frIs7[nlp-29p*::*gfp*, *col-12p*::*DsRed]* [20]. Worms were transferred onto RNAi plates at the L1 stage.

### Infection, wounding, osmotic stress and DHCA treatment

Infections, epidermal wounding and osmotic stress or dihydrocaffeic acid (DHCA) treatments were performed as previously described [10, 11, 20, 25].

### Killing and longevity assays

For the experiments reported in Figs 3 and S2C, 50–70 worms at the L1 stage were cultured on the appropriate RNAi bacterial clone at 25°C, and then (for Fig 3) infected at the young adult stage for 1h with *D*. *coniospora* and transferred to fresh RNAi plates and cultured at 15°C (to accentuate differences in survival [61]), or transferred directly to fresh RNAi plates and cultured at 20°C (for S2C Fig). In both cases, the surviving worms were counted every day as described elsewhere [62]. For the experiments reported in Fig 7, worms at the L1 stage were cultured on the appropriate RNAi bacterial clone at 25°C for 32 hours and then infected with *D*. *coniospora* overnight. Groups of 20–30 worms were then transferred to wells in 12-well plates (3 wells per condition), and images of each well collected automatically at regular intervals (roughly every 20 minutes) using a custom system that will be described elsewhere. The images were then examined, and worms scored as dead when they no longer showed sign of any movement between images. Statistical analyses used one-sided log rank test within Prism (Graphpad software).

### Analyses with the Biosort worm sorter

Expression of *nlp-29p*::*gfp* reporter was quantified with the COPAS Biosort (Union Biometrica). Generally, a minimum of 80 synchronized worms were analyzed for size (TOF), extinction (EXT), green (GFP) and red (dsRed) fluorescence. The ratio Green/TOF was then calculated to normalize the fluorescence. When only mean values for ratios are presented, the values for the different samples within a single experiment are normalized so that the control worms (WT) had a ratio of 1. As discussed more extensively elsewhere [38], standard deviations are not an appropriate parameter and are not shown on figures with the Biosort. The results shown are representative of at least 3 independent experiments.

### RNA preparation and quantitative RT -PCR

RNA preparation and quantitative RT-PCR were done as described [24]. Results were normalized to those of *act-1* and were analyzed by the cycling threshold method. Control and experimental conditions were tested in the same ‘run’. Each sample was normalized to its own *act-1* control to take into account age-specific changes in gene expression.

### qRT-PCR primers

Primers used for qRT-PCR are for:

*act-1*: JEP538 ccatcatgaagtgcgacattg JEP539 catggttgatggggcaagag;

*dcar-1*: JEP2030 cctacgctatttggtgcattggct JEP2031 tgcaccgaatcaccagaaacag;

*nlp-27*: JEP965 cggtggaatgccatatggtg JEP966 atcgaatttactttccccatcc;

*nlp-28*: JEP967 tatggaagaggttatggtgg JEP968 gctaatttgtctactttcccc;

*nlp-29*: JEP952 tatggaagaggatatggaggatatg JEP848 tccatgtatttactttccccatcc;

*nlp-30*: JEP948 tatggaagaggatatggtggatac JEP949 ctactttccccatccgtatcc;

*nlp-31*: JEP950 ggtggatatggaagaggttatggag JEP953 gtctatgcttttactttcccc;

*nlp-34*: JEP969 atatggataccgcccgtacg JEP970 ctattttccccatccgtatcc;

### Affinity co-purification assays

Affinity co-purification assays were performed as previously described [63] with minor modifications. From 3 independent mixed stage cultures of control *rde-1(ne300)* or *rde-1(ne300); akir-1(gk528)* worms carrying *akir-1p*::*AKIR-1*::*gfp*, samples were harvested, yielding about 4 g of flash-frozen pellets of *C*. *elegans*. In parallel, samples were also prepared from equivalent cultures that had been infected with *D*. *coniospora* for 16 h at 25°C. Frozen samples were defrosted in a presence of lysis buffer (0.1% Nonidet P-40 Substitute, 50 mM Tris/HCl, pH 7.4, 100 mM KCl, 1 mM MgCl_2_, 1 mM EGTA pH 8.0, 10% glycerol, protease inhibitor cocktail (Roche), 1 mM DTT) and sonicated on Diagenode (cycle: 0.5 s, amplitude: 40–45%, 10 sessions, interval between sessions: 30 s). After sonication, Nonidet P-40 Substitute was added up to 1% and the lysates were incubated with head over tail rotation at 4°C for 30 min, followed by centrifugation at 20,000° g for 20 min at 4°C. Cleared lysate was then collected and split into either the anti-GFP agarose beads or the blocked control beads (40–50 μl, NanoTrap, Chromotek) (Fig 5A). After head over tail rotation at 4°C for 60–90 min, the beads were washed once with lysis buffer containing 0.1% Nonidet P-40 Substitute, followed by two washings in each of the buffers I (25 mM Tris-HCl, pH 7.4, 300 mM NaCl, 1 mM MgCl_2_) then buffer II (1 mM Tris-HCl, pH 7.4, 150 mM NaCl, 1 mM MgCl_2_). Proteins were eluted twice by orbital shaking in 100 μl of 6 M urea followed by ethanol precipitation. Precipitated proteins were resolubilized in 6 M urea/2 M thiourea buffer (10 mM HEPES, pH 8.0). Reduction and alkylation of proteins were then performed at room temperature, followed by digestion in solution sequentially using lysyl endopeptidase (Lys-C, Wako) for 3 h and trypsin (Promega) overnight as previously described [64]. Peptides were purified by solid phase extraction in C18 StageTips [65].

### Immunoprecipitation assay

Mixed stage worms (IG1665) carrying AKIR-1::GFP and CEH-18::GFP::FLAG were harvested on ice and lysed in lysis buffer (0.5% Nonidet P-40 Substitute, 50 mM Tris/HCl, pH 7.4, 100 mM KCl, 1 mM MgCl_2_, 1 mM EGTA, 10% glycerol, protease and phosphatase inhibitor cocktail (Roche), 1 mM DTT), subjected to three cycles of freeze and thaw and sonicated on Diagenode (cycle: 0.5 s, amplitude: high, 5 min, interval between sessions: 30 s). Lysates were cleared by centrifugation. 200 μg of total protein was used for each immunoprecipitation: with anti-Flag (M2 clone, Sigma), and anti-HA as the unrelated control antibody (clone HA.11). Co-immunobound proteins were precipitated using Dynabeads Protein G matrix (ThermoFisher) and eluted in SDS buffer (1% SDS in TE, 150 mM NaCl). Immunoprecipitates were then resolved on a gel and subjected to Western blot analysis as described below.

### Liquid chromatography tandem mass spectrometry

Peptides were separated in an in-house packed analytical column (inner diameter: 75 μm; ReproSil-Pur C18-AQ 3-μm resin, Dr. Maisch GmbH) by online nanoflow reversed phase chromatography through an 8–50% gradient of acetonitrile with 0.1% formic acid (120 min). The eluted peptides were sprayed directly by electrospray ionization into a Q Exactive Plus Orbitrap mass spectrometer (Thermo Scientific). Mass spectrometry measurement was carried out in data-dependent acquisition mode using a top10 sensitive method with one full scan (resolution: 70,000, target value: 3 × 10^6^) followed by 10 fragmentation scans via higher energy collision dissociation (HCD; resolution: 35,000, target value: 5 × 10^5^, maximum injection time: 120 ms, isolation window: 4.0 m/z). Precursor ions of unassigned or +1 charge state were rejected for fragmentation scans. Dynamic exclusion time was set to 30 s.

### Mass spectrometry data analysis

Raw data files were processed by MaxQuant software package (version 1.5.5.0) [66] using Andromeda search engine [67]. Spectral data were searched against a target-decoy database consisting of the forward and reverse sequences of WormPep release WS254 (28,071 entries), UniProt *E*. *coli* K-12 proteome release 2016_02 (4,314 entries) and a list of 245 common contaminants. Trypsin/P specificity was selected. Carbamidomethylation of cysteine was chosen as fixed modification. Oxidation of methionine and acetylation of the protein N-terminus were set as variable modifications. A maximum of 2 missed cleavages were allowed. The minimum peptide length was set to be 7 amino acids. At least one unique peptide was required for each protein group. False discovery rate (FDR) was set to 1% for both peptide and protein identifications.

Protein quantification was performed using the LFQ label-free quantification algorithm [68]. Minimum LFQ ratio count was set to one. Both the unique and razor peptides were used for protein quantification. The “match between runs” option was used for transferring identifications between measurement runs allowing a maximal retention time window of 0.7 min. All raw mass spectrometry data have been deposited in the PRIDE repository with the dataset identifier PXD008074.

Statistical data analysis was performed using R statistical software. Only proteins quantified in at least two out of the three GFP pull-down replicates (or two out of two GFP pull-downs for the experiment using infected worms) were included in the analysis. LFQ intensities were log2-transformed. Imputation for missing values was performed for each pull-down replicate in Perseus [69] software (version 1.5.5.0) using a normal distribution to simulate low intensity values below the noise level (width = 0.3; shift = 1.8). The LFQ abundance ratio was then calculated for each protein between the GFP pull-downs and the controls. Significance of the enrichment was measured by an independent-sample Student's *t* test assuming equal variances. Specific interaction partners were then determined in a volcano plot where a combined threshold (hyperbolic curve) was set based on a modified *t*-statistic (*t*(SAM, significance analysis of microarrays); *s*_*0*_ = 1.5, *t*_*0*_ = 0.9 ∼ 1.5) [70, 71]. Proteins cross-reactive to the anti-GFP antibody were identified by a pull-down experiment using the non-transgenic *rde-1* strain and were filtered out from the AKIR-1 protein interactor dataset.

### Western blot analysis

Samples for western blot analysis were either prepared as per the co-precipitation protocol with the final elution performed in 50 μl 200 mM glycine pH 2.6 and immediately neutralisation by addition of 0.2 M Tris pH 10.4, or as per the immunoprecipitation protocol. Samples were then resolved on a 4–12% BisTris Gel (Invitrogen) and subjected to transfer to a membrane.

Primary antibodies used in that study were as follow: anti-GFP (clone 11E5, Invitrogen, dilution 1:2000), anti-HDA-1 (Santa Cruz, dilution 1:2000), anti-LET-418 (kind gifts of F. Muller and C. Wicky, used at 1:500), anti-FLAG (M2, Sigma, dilution 1:2000) and anti-actin (Abcam, dilution 1:1500). The membrane was then incubated with horseradish peroxidase-conjugated secondary antibodies (1:10,000) at room temperature for 1 h, followed by brief incubation with substrates for enhanced chemiluminescence (Pierce ECL Plus).

### Chromatin immunoprecipitation

For extract preparations, N2 worms were grown on rich NGM seeded with HT115 bacteria, and young adult populations of worms were used to prepare about 3–4 gr of flash frozen worm popcorn. Worms were then fixed first with 1.5 mM EGS (ethylene glycol bis) for 20 min and then in 1.1% formaldehyde, with protease and phosphatase inhibitors, at room temperature with shaking, for 20 min. The fixing reaction was quenched by addition of glycine to a final concentration of 125 mM. Worms were then washed once with 10 ml FA buffer (50 mM HEPES/KOH (pH 7.5), 1 mM EDTA, 1% Triton X-100, 0.1% sodium deoxycholate, 150 mM NaCl) with protease inhibitors (Pierce), resuspended in FA buffer containing 0.1% sarkosyl and protease and phosphatase inhibitors, then dounce-homogenized on ice. Well-resuspended mixtures were then sonicated to shear chromatin (size rage 300–800 bp) using 12 cycles (30’ on, 30’ off) in a Bioruptor-Pico (Diagenode). Cellular debris was removed by centrifugation at 17,000 *g* for 15 min at 4°C. Immunoprecipitation reactions contained approximately 3 mg of total protein, with 1% sarkosyl. Before addition of the antibody (NanoTrap-GFP, Chromotek), 5% of the material was taken as input. Immunocomplexes were collected and washed with 1 ml of the following buffers: FA buffer, two washes, 5 min each; FA buffer with 1 M NaCl, 5 min; FA with 500 mM NaCl, 10 min; TEL buffer (0.25 M LiCl, 1% NP-40, 1% sodium deoxycholate, 1 mM EDTA, 10 mM Tris-HCl, pH 8.0), 10 min, and TE (pH 8.0), two washes, 5 min each. Complexes were eluted in 1% SDS in TE with 250 mM NaCl at 65°C for 30 min. Samples and inputs were treated with Proteinase K for 1 h, and cross-links were reversed at 65°C overnight. DNA was purified with Qiagen PCR purification columns. Locus-specific ChIP qPCR reactions (SYBR Premix ExTaq II, TaKara) were done for each immunoprecipitation using specific elution (ChIP), negative control elution (nonspecific) and input samples, following a 50-fold dilution. Ct values were used to calculate the fold difference in DNA concentration between ChIP and nonspecific samples, normalized to the input.

*p_act-1*^A^: JEP2537 gggcgggtcaaacagaaa, JEP2538 atgcgccgcccttttatt

*p_act-1*^B^ JEP2522 tgcaagtgcagcgagaaa, JEP2528 aacacgttcgtcgcgttg

*p_nlp-29*: JEP2521 gaaaaagaaacagagtctcgtgatg, JEP2527 tttctgattattaccacgtttttcg

*p_nlp-31*: JEP2529 cccagttcttcgtgtcaccac, JEP2530 gccgggcaaaatcacaaa

*p_nlp-34*: JEP2535 gacgtacctagacgtagaccatacacc, JEP2536 gtgacgtaattcgcaacatgg

*3’UTR_nlp-29*: JEP2544 ggggaagaaaataatttacatgagc, JEP2545 gcaagcgcaaaaatgttaaaaa

*3’UTR_nlp-31*: JEP2531 gcttttaataatatgacatgaccgaaa, JEP2532 gaaatttgacattcatcaaaatgct

*3’UTR_nlp-34*: JEP2539 ccgtacggatacggaggata, JEP2540 tttaaagtatattcgtcagcagcag

### Microscopy

Confocal images were captured using an inverted confocal spinning disk microscope (Yokogawa, Visitron Systems GmbH) associated with a 512 x 512 pixels EM-CCD camera (Hamamatsu). Worms were immobilized in 0.01% levamisole and visualized through a CFI Plan Fluor Nikon 40X oil, 1.3 NA objective and 1.5X lens, using a 488 nm laser. Z-stacks were acquired with a step size of 0.3 μm.

## Supporting information

S1 Fig Akirin controls the expression of *nlp-29*, an AMP encoding gene. **A.** Quantification in arbitrary, but constant units of relative size (Time Of Flight; TOF; grey bars), optical density (Extinction; Ext; orange bars) and *col-12p*::*dsRed* expression (red bars) of wild type worms carrying the integrated array *frIs7* (which contains the fluorescent reporter transgenes *nlp-29p*::*gfp* and *col-12p*::*DsRed*) treated with RNAi against *sta-1*, *dcar-1* and *akir-1*. In all cases there are no significant differences between control and experimental values (paired two-sided student t test). **B.** Ratio of green fluorescence (GFP) to size (TOF) of wild type and *akir-1(gk528)* worms carrying *frIs7* and assessed without further treatment (control) or after infection by *D*. *coniospora*. Data are representative of three independent experiments. **C.** Comparisons of growth (left panel), *DsRed* expression (middle panel) and optical density (right panel) between wild type and *akir-1(gk528)* worms carrying *frIs7* on 3 successive days after hatching. Data are representative of three independent experiments. A minimum of 50 worms was analysed for each condition. **D.** Quantification of *D*. *coniospora* spore adhesion at the level of the nose and the vulva in wild type worms carrying the *frIs7* array and treated with RNAi against *sta-1*, *dcar-1* and *akir-1*.
(PDF)

S2 Fig Akirin controls *nlp-29* expression in the epidermis and longevity. **A.** Abundance of mRNA for genes in the *nlp-29* cluster in *rde-1*(*ne219*); *wrt-2p*::*RDE-1* worms treated with RNAi against *sta-1*, *dcar-1* or *akir-1*, presented as the difference in cycling threshold (ΔCt) between each *nlp* gene and *act-1*. Data are from three independent experiments (average and SD). **B.** Fold induction of expression for *nlp* genes in each of 3 experiments in *rde-1*(*ne219*); *wrt-2p*::*RDE-1* worms treated with RNAi against the indicated genes and infected by *D*. *coniospora*; results are presented relative to those of uninfected worms. The average values are represented in Fig 2C. **C.** Survival of *rde-1*(*ne219*);*wrt-2p*::*RDE-1* worms treated with RNAi against *sta-1* (n = 50) or *akir-1* (n = 50). The difference between the *sta-1(RNAi)* and *akir-1(RNAi)* animals is highly significant (p<0.0001; one-sided log rank test). Data are representative of three independent experiments.
(PDF)

S3 Fig *nlp-29* expression is independent of the SWI/SNF nucleosome remodelling complex. Ratio of green fluorescence (GFP) to size (TOF) (**A**) and quantification in arbitrary, but constant units of relative size (TOF; grey bars), *col-12p*::*dsRed* expression (red bars) and optical density (Ext; orange bars) in wild-type worms carrying *frIs7* and treated with RNAi against different genes (**B, C**). The genes corresponding to core (*swsn-1*, *swsn-4*, *swsn-5*), accessory (*swsn-2*.*1*, *swsn-3*, *dpff-1*), PBAF/PBAP (*swsn-7 and pbrm-1*) and BAF/PBAF (*let-526 and swsn-9*) elements of the SWI/SNF nucleosome remodelling complex are indicated. Populations of >100 worms were analysed for each condition. Data are representative of three independent experiments. **D.** Quantification of *D*. *coniospora* spore adhesion at the level of the nose and the vulva in wild type worms carrying *frIs7* and treated with RNAi against the indicated genes.
(PDF)

S4 Fig LET-418 NuRD and MEC complexes act to modulate *nlp* AMP gene expression in the epidermis. **A.** Ratio of green fluorescence (GFP) to size (TOF) in *rde-1*(*ne219*);*wrt-2p*::*RDE-1* worms that are largely resistant to RNAi except in the epidermis carrying the array *frIs7* treated with RNAi against the indicated genes and then infected or not by *D*. *coniospora*. **B.** Quantitative RT-PCR analysis of the fold induction of expression of genes in the *nlp-29* cluster in *rde-1*(*ne219*); *wrt-2p*::*RDE-1* worms treated with RNAi against the indicated genes, comparing expression levels in worms infected by *D*. *coniospora* with uninfected worms. The 6 RNAi clones block the induction of expression of each of the endogenous *nlp* AMP genes more or less completely, with the exception of *nlp-28*. Data are from three independent experiments (average and SD).
(PDF)

S5 Fig Transgenic rescue of *akir-1* mutant phenotypes. **A.** Quantitative RT-PCR analysis of the fold induction of expression of genes in the *nlp-29* cluster in *rde-1*(*ne219*), *rde-1*(*ne219*);*akir-1*(*gk528*) and *rde-1*(*ne219*);*akir-1*(*gk528*); *akir-1p*::*AKIR-1*::*gfp* worms, comparing expression levels in worms infected by *D*. *coniospora* with uninfected worms. Data are from three independent experiments (average and SD). **B.** Lifespan of *rde-1*(*ne219*), *rde-1*(*ne219*);*akir-1*(*gk528*) and *rde-1*(*ne219*);*akir-1*(*gk528*); *akir-1p*::*AKIR-1*::*gfp* worms. Data are representative of three independent experiments.
(PDF)

S6 Fig Expression pattern of AKIR-1::GFP. Worms carrying a single copy insertion of an AKIR-1::GFP construct (*wt; frSi12[pNP157(akir-1p*::*AKIR-1*::*GFP)] II*) were visualized by confocal microscopy and several confocal planes summed. AKIR-1::GFP showed a clear and strong nuclear localization: **A.** epidermal nuclei in an L3 stage worm **B.** Germline nuclei in a young adult. The scale bar is 20 μm.
(PDF)

S1 Table Identification of protein-protein interactors for AKIR-1. The quantitative results for analyses of 3 independent samples are given, referenced to Wormbase release WS254.
(XLSX)

S2 Table Comparison of protein-protein interactors for AKIR-1 and SWSN-2.2. Data was from [34] and S1 Table. The annotations come from Wormbase (WS257). Gene identifiers were made uniform using Wormbase Converter [37].
(XLSX)

S3 Table Raw data for figures. The numerical data underlying the different figures is presented in individual sheets, arranged by category (Biosort, survival and qRT-PCR).
(XLSX)
