# Supplementary figures and images for: Evolutionary plasticity in the innate immune function of Akirin
Source: PLoS Genet. 2018 Jul 23;14(7):e1007494. doi: 10.1371/journal.pgen.1007494 (PMC6072134; doi:10.1371/journal.pgen.1007494)

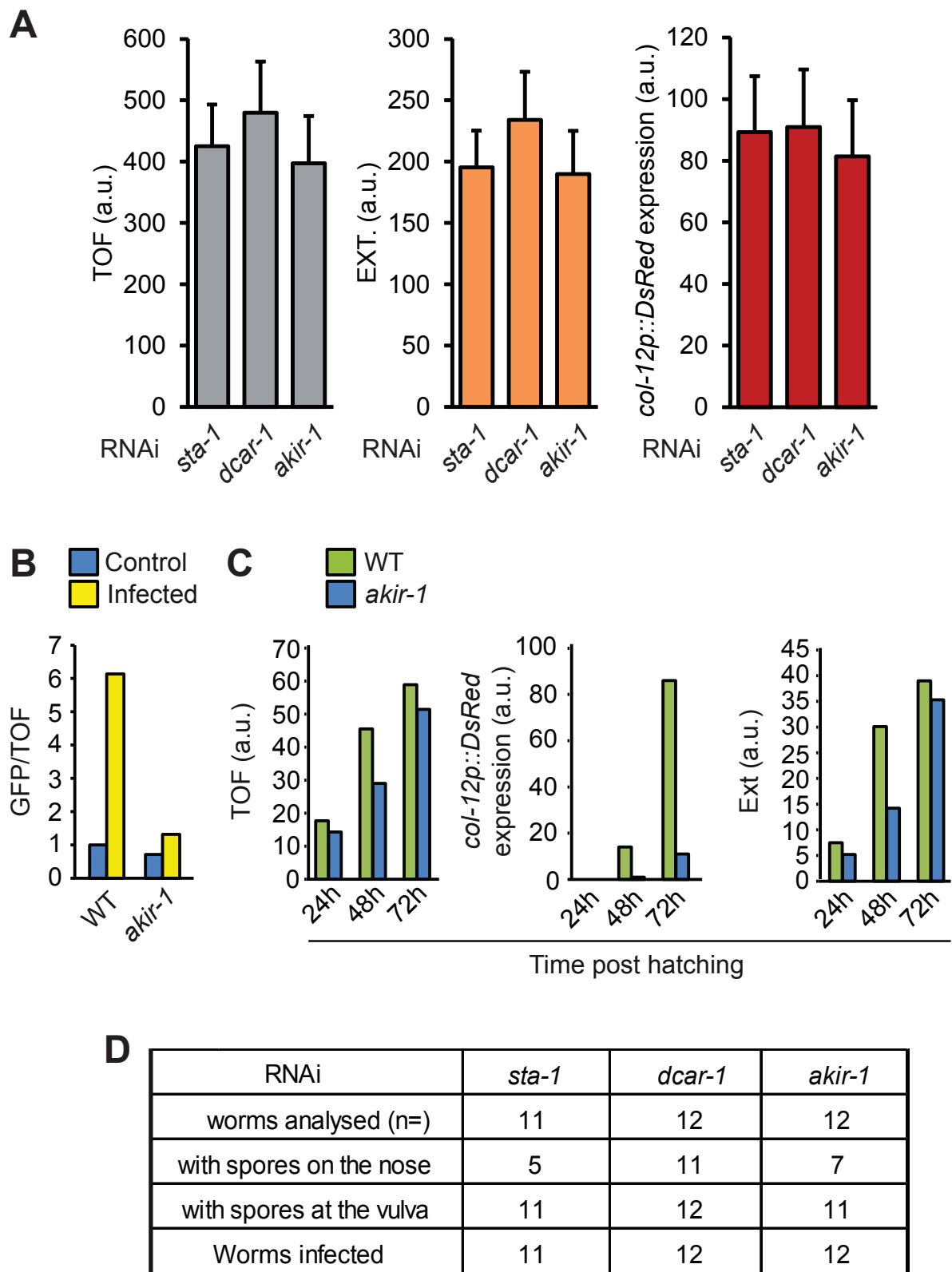

Figure S1

Supplement: S1 Fig — A. Quantification in arbitrary, but constant units of relative size (Time Of Flight; TOF; grey bars), optical density (Extinction; Ext; orange bars) and col-12p::dsRed expression (red bars) of wild type worms carrying the integrated array frIs7 (which contains the fluorescent reporter transgenes nlp-29p::gfp and col-12p::DsRed) treated with RNAi against sta-1, dcar-1 and akir-1. In all cases there are no significant differences between control and experimental values (paired two-sided student t test). B. Ratio of green fluorescence (GFP) to size (TOF) of wild type and akir-1(gk528) worms carrying frIs7 and assessed without further treatment (control) or after infection by D. coniospora. Data are representative of three independent experiments. C. Comparisons of growth (left panel), DsRed expression (middle panel) and optical density (right panel) between wild type and akir-1(gk528) worms carrying frIs7 on 3 successive days after hatching. Data are representative of three independent experiments. A minimum of 50 worms was analysed for each condition. D. Quantification of D. coniospora spore adhesion at the level of the nose and the vulva in wild type worms carrying the frIs7 array and treated with RNAi against sta-1, dcar-1 and akir-1. (PDF) [file pgen.1007494.s001.pdf]

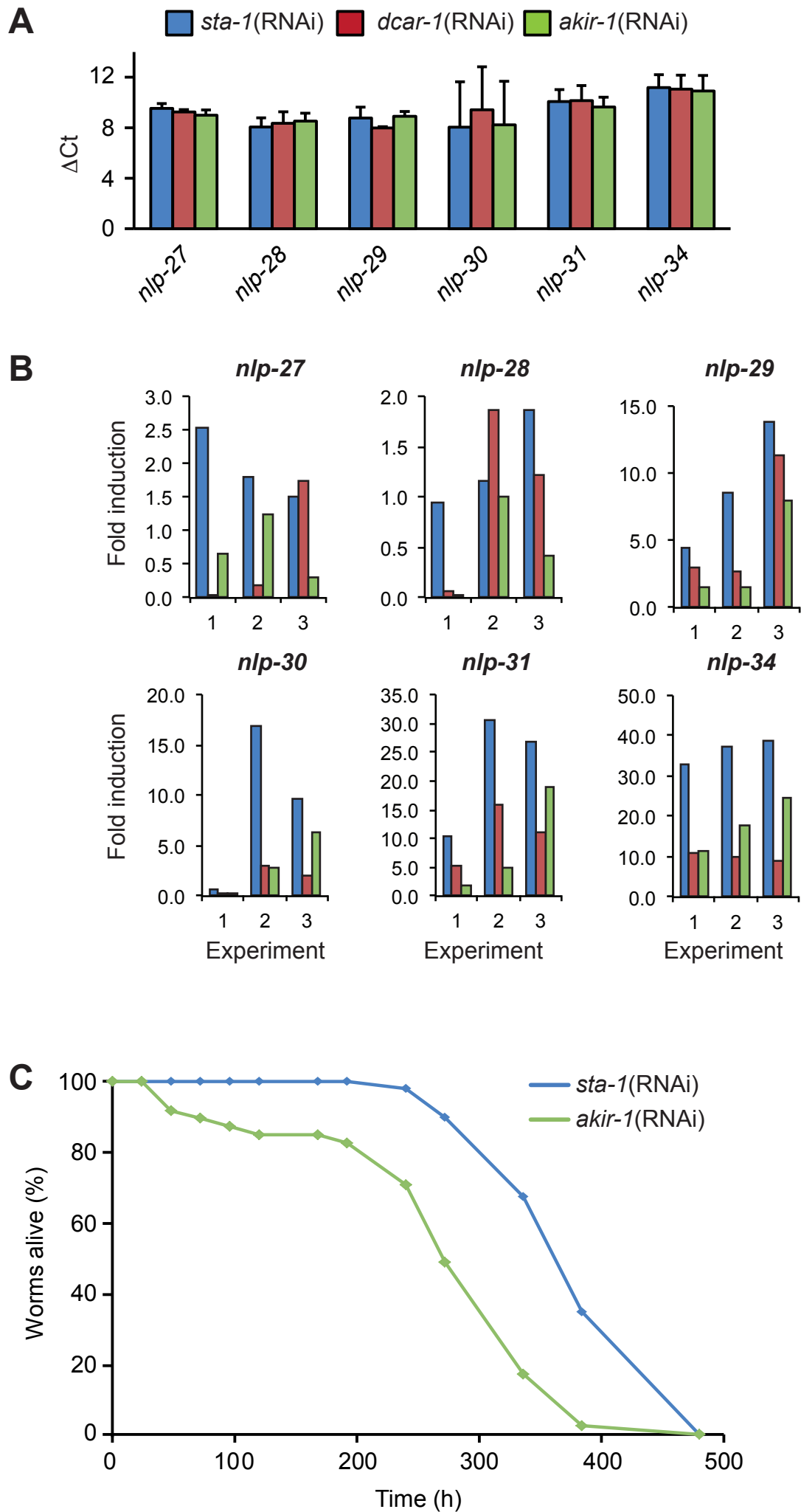

Figure S2

Supplement: S2 Fig — A. Abundance of mRNA for genes in the nlp-29 cluster in rde-1(ne219); wrt-2p::RDE-1 worms treated with RNAi against sta-1, dcar-1 or akir-1, presented as the difference in cycling threshold (ΔCt) between each nlp gene and act-1. Data are from three independent experiments (average and SD). B. Fold induction of expression for nlp genes in each of 3 experiments in rde-1(ne219); wrt-2p::RDE-1 worms treated with RNAi against the indicated genes and infected by D. coniospora; results are presented relative to those of uninfected worms. The average values are represented in Fig 2C. C. Survival of rde-1(ne219);wrt-2p::RDE-1 worms treated with RNAi against sta-1 (n = 50) or akir-1 (n = 50). The difference between the sta-1(RNAi) and akir-1(RNAi) animals is highly significant (p<0.0001; one-sided log rank test). Data are representative of three independent experiments. (PDF) [file pgen.1007494.s002.pdf]

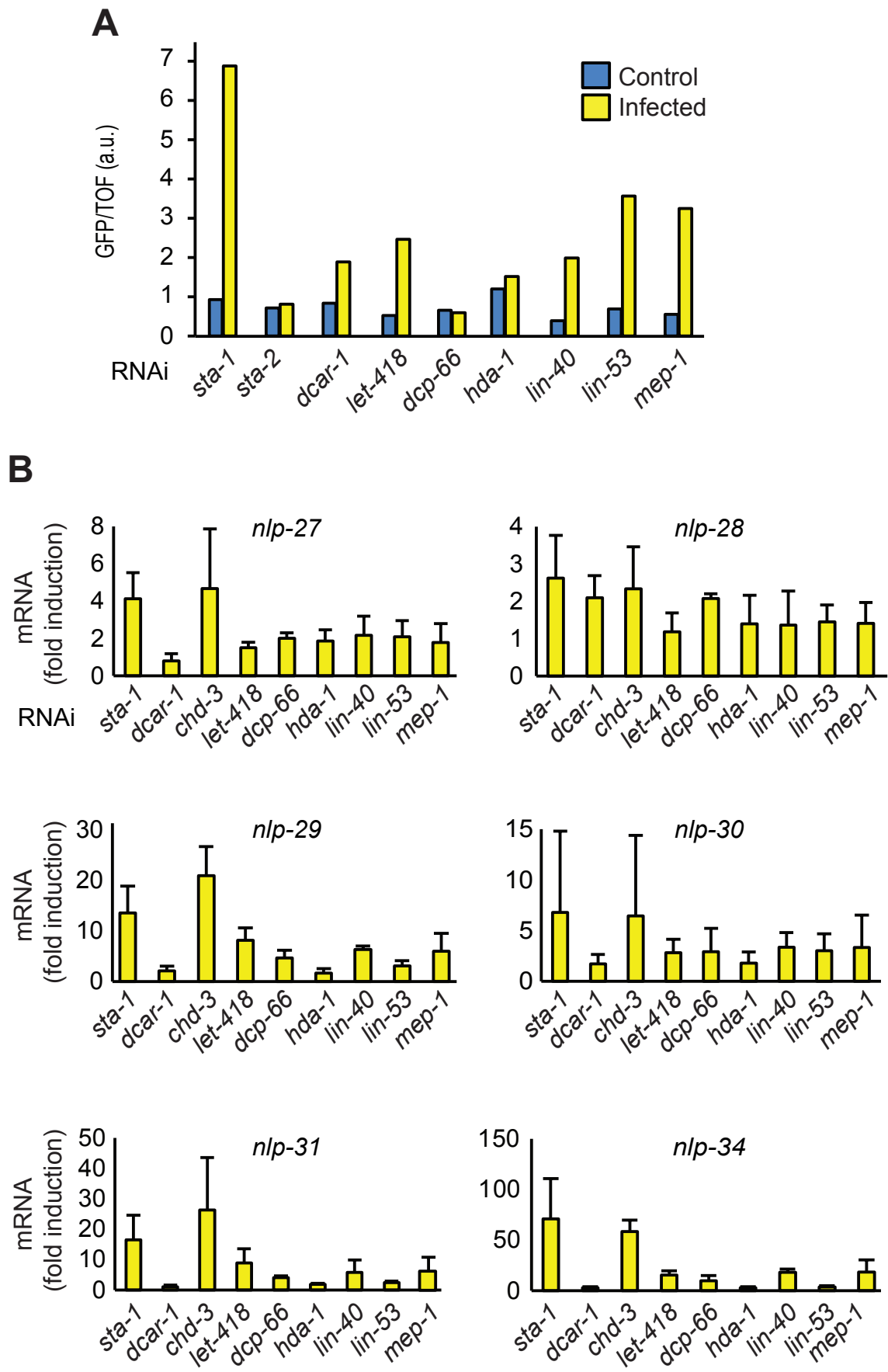

Fig S4

Supplement: S4 Fig — A. Ratio of green fluorescence (GFP) to size (TOF) in rde-1(ne219);wrt-2p::RDE-1 worms that are largely resistant to RNAi except in the epidermis carrying the array frIs7 treated with RNAi against the indicated genes and then infected or not by D. coniospora. B. Quantitative RT-PCR analysis of the fold induction of expression of genes in the nlp-29 cluster in rde-1(ne219); wrt-2p::RDE-1 worms treated with RNAi against the indicated genes, comparing expression levels in worms infected by D. coniospora with uninfected worms. The 6 RNAi clones block the induction of expression of each of the endogenous nlp AMP genes more or less completely, with the exception of nlp-28. Data are from three independent experiments (average and SD). (PDF) [file pgen.1007494.s004.pdf]

**A**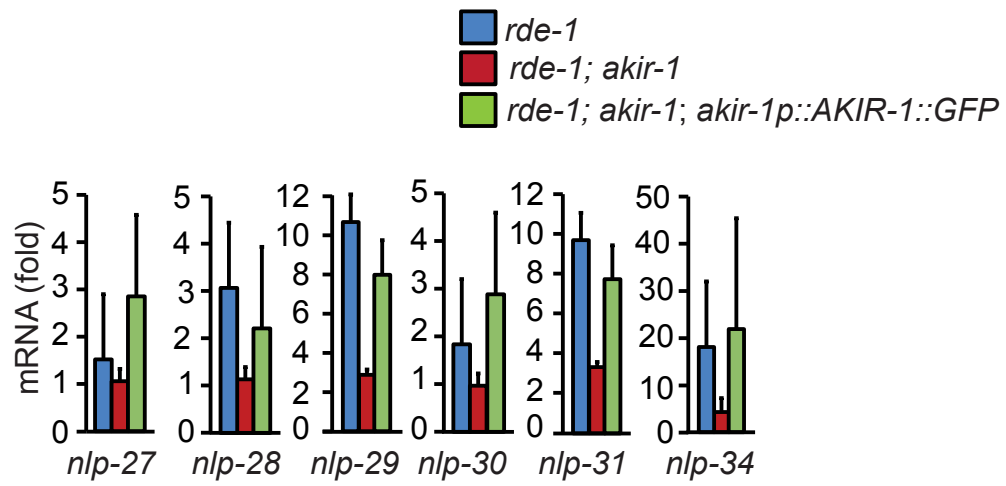**B**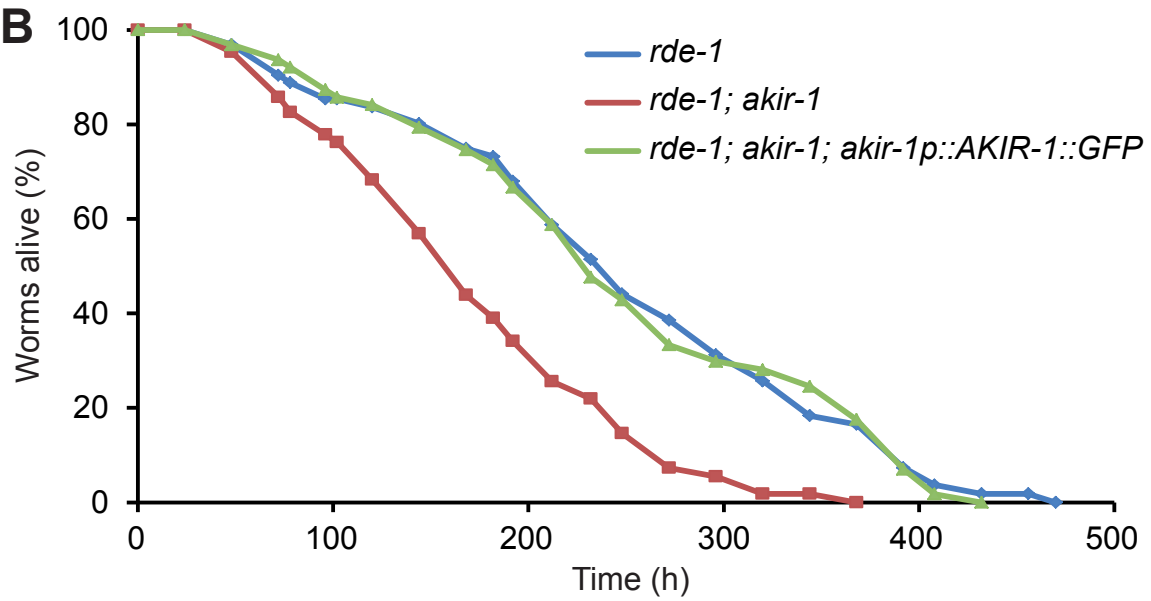

Figure S5

Supplement: S5 Fig — A. Quantitative RT-PCR analysis of the fold induction of expression of genes in the nlp-29 cluster in rde-1(ne219), rde-1(ne219);akir-1(gk528) and rde-1(ne219);akir-1(gk528); akir-1p::AKIR-1::gfp worms, comparing expression levels in worms infected by D. coniospora with uninfected worms. Data are from three independent experiments (average and SD). B. Lifespan of rde-1(ne219), rde-1(ne219);akir-1(gk528) and rde-1(ne219);akir-1(gk528); akir-1p::AKIR-1::gfp worms. Data are representative of three independent experiments. (PDF) [file pgen.1007494.s005.pdf]

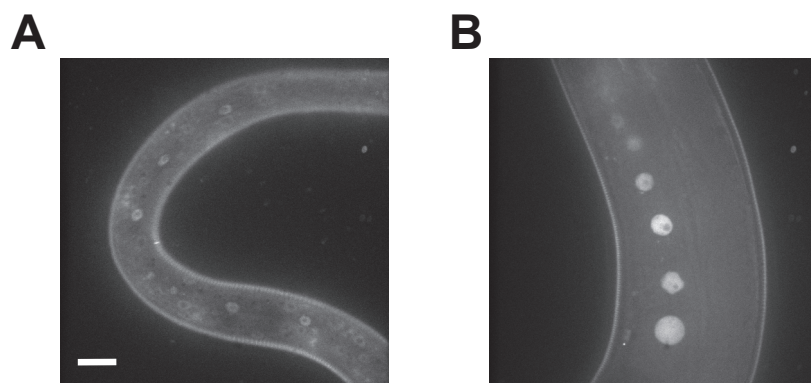

Figure S6

Supplement: S6 Fig — Worms carrying a single copy insertion of an AKIR-1::GFP construct (wt; frSi12[pNP157(akir-1p::AKIR-1::GFP)] II) were visualized by confocal microscopy and several confocal planes summed. AKIR-1::GFP showed a clear and strong nuclear localization: A. epidermal nuclei in an L3 stage worm B. Germline nuclei in a young adult. The scale bar is 20 μm. (PDF) [file pgen.1007494.s006.pdf]
