# Supplementary material for: Evolutionary plasticity in the innate immune function of Akirin
Source: PLoS Genet. 2018 Jul 23;14(7):e1007494. doi: 10.1371/journal.pgen.1007494 (PMC6072134; doi:10.1371/journal.pgen.1007494)
Supplement: S3 Fig — Ratio of green fluorescence (GFP) to size (TOF) (A) and quantification in arbitrary, but constant units of relative size (TOF; grey bars), col-12p::dsRed expression (red bars) and optical density (Ext; orange bars) in wild-type worms carrying frIs7 and treated with RNAi against different genes (B, C). The genes corresponding to core (swsn-1, swsn-4, swsn-5), accessory (swsn-2.1, swsn-3, dpff-1), PBAF/PBAP (swsn-7 and pbrm-1) and BAF/PBAF (let-526 and swsn-9) elements of the SWI/SNF nucleosome remodelling complex are indicated. Populations of >100 worms were analysed for each condition. Data are representative of three independent experiments. D. Quantification of D. coniospora spore adhesion at the level of the nose and the vulva in wild type worms carrying frIs7 and treated with RNAi against the indicated genes. (PDF) [file pgen.1007494.s003.pdf]

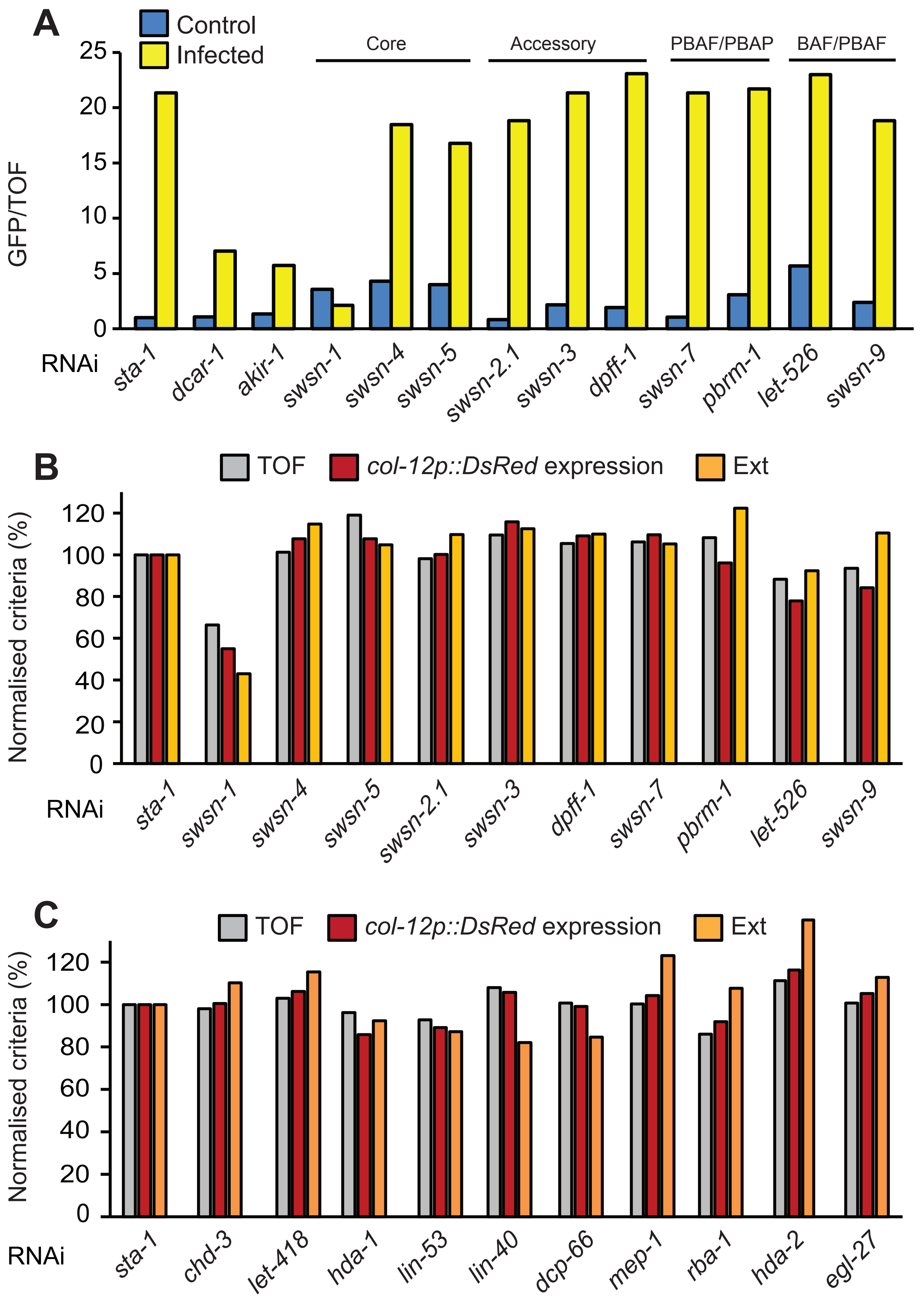

D

RNAi	*sta-1*	*chd-3*	*let-418*	*hda-1*	*lin-53*	*lin-40*	*dcp-66*	*mep-1*	*rba-1*	*egl-27*	*hda-2*
Worms analysed (n=)	11	12	10	10	10	12	13	12	10	10	11
with spores on the nose	5	8	5	8	8	9	11	10	8	7	7
with spores on the vulva	11	12	10	10	10	12	13	12	10	10	11
Worms infected	11	12	10	10	10	12	13	12	10	10	11

Figure S3
